# Supplementary material for: Impact of Long Covid on the school experiences of children and young people: a qualitative study
Source: BMJ Open. 2023 Sep 19;13(9):e075756. doi: 10.1136/bmjopen-2023-075756 (PMC10510910; doi:10.1136/bmjopen-2023-075756)
Supplement: Supplementary data [file bmjopen-2023-075756supp001.pdf]

Supplementary file – interview guides

Interview Guide for Experiences of Long Covid in Families study – parents & young people who have had Long Covid themselves (patients)

NB. Participants may occupy dual roles and be both ***carers*** *and* ***patients****. Topic guide can be modified accordingly.*

PREAMBLE for young people (who have had Long Covid themselves)

PART ONE: INVITATION TO TELL THEIR OWN STORY OF LONG COVID IN THE FAMILY

In this interview, we would like to hear about your experiences of how Long Covid has affected you and your family. I am interested in whatever you would like to tell me about your experiences (e.g. how this affected where you were living and working/studying and any other health problems you were dealing with) but please do feel free to take a break whenever you need to – we can continue another day if you get tired.

In the first part of the interview I will ask you to tell me about your experiences. After you have finished your story, I will ask you a few extra questions if that's OK.

PART TWO: FOLLOW-UP PROMPTS TO COVER ANY TOPICS NOT COVERED IN INTERVIEWEE'S NARRATIVE - PARENTS AND YOUNG PEOPLE

Initial awareness of Long Covid and ideas about own/ family vulnerability

The purpose of this area of questioning is to develop an understanding of how Long Covid became a concern for the participant and their family and if they made any changes in response (beyond those already in place around Covid)

First awareness of Long Covid

When did you first hear about Long Covid? Can you remember how you heard about it and what you thought it meant? Where did your information come from?

Did you have particular worries or concerns about yourself or members of your family and those close to you?

For example, did you hear anything in the media about some groups of people being more affected by Long Covid than others? How did you feel about that?

Did the awareness of Long Covid lead you to you make any additional changes (i.e. changes beyond those you might have already made in relation to COVID-19) to your daily life or to the people you would see?

Did other people in your family make changes as well? How did that work?

Experiences of becoming ill with Long Covid

Suspecting Long Covid

When did you realise that you might have Long Covid? *(NB use terminology that participant has used to this point)* What was your life like at that time? (school life, work life, social life, family life)

Which month and year was it when you had COVID-19, and then when did you realise it was Long Covid?

If diagnosed: How did you feel about getting diagnosed with Long Covid? Has this changed over time?

Illness experience

(How) has your experience of having Long Covid changed over time? (Prompts: *How) have the symptoms changed over time? Are there any triggers that make your symptoms worse? Is there anything that helps with your symptoms? What have you tried? What happened when you tried this? What has been suggested by others? Are there any symptoms that particularly worry you – either now or thinking about the longer term?*)

(*If relevant – may have been previously fit and well*) Thinking about the health problems or any health conditions you had before you developed Long COVID, have any of these have changed (got worse/better)

Who did you tell when you first had/thought you had Long Covid? (did you tell people you have contact with regularly? What were their responses?)

Actions taken

Have you been able to take care of yourself when you were ill? Did anyone else help care for you?

How did you do to manage in your day-to-day life when you developed Long Covid? Did you make any changes to your usual routines? Did any family members have to make changes to their daily life?

Were there important things that you weren't able to do while you were ill (work, caring responsibilities, religious activities)? How did you manage those things?

Family life

How has long covid affected your day-to-day life? (*prompts: work, relationships, ability to do things you previously enjoyed, sleep etc*)

Can you tell me about how Long Covid has affected your family life?

Have there been any disruptions to key tasks in your family life e.g. to childcare, household routines, work, shopping, family-owned or run businesses etc.

If split or multiple households: can you tell me about how you've managed across households? How about with extended family?

Have there been any changes in your family roles? (*prompts: who works, childcare, household tasks)*

What was your day like yesterday? OR Talk me through what a typical day looks like for your family. Have there been times when it was more difficult? What has made it easier?

Have you had to deal with anything similar to this before as a family? *If yes:* can you tell me about your earlier experiences?

If relevant: Do you have any concerns about regaining your independence / regaining normality?

Is there anything you continue to struggle with as a family? How does this make you feel about the future?

Are there any other ways in which having Long Covid is still affecting your day-to-day family life or your plans for the future? (use prompts as necessary)

- Impact on work school and other daily activities
- Impact on family and social life
- Returning to work/getting a new job (support from employer/colleagues, Occupational Health, adjustments, changed hours and positive and negative experiences)
- Financial issues (were you working, furloughed, did you get sick leave, did you get sick pay, state benefits, travel costs, insurances, change in outgoings, for younger people: statement of special educational needs, disabled students allowance)
- Things you've had to cancel, abandon or postpone

Was there anything you felt was particularly unfair about your family's experience of Long Covid?

Information and care-seeking

How was the communication with your GP? What went well? What didn't go well? Talk me through what the conversation was like.

Can we talk a bit more about info about LC?

What are the main sources of information that you have accessed or made use of in relation to Long Covid? What if anything has been helpful/unhelpful about this information?

To what extent do you think Long Covid is well understood? (by those around you, healthcare staff, employers)

Accessing healthcare for LC
How did you come to understand that you have LC?
Have you seen your GP about your LC?

Can you talk me through how you've found accessing healthcare for LC?

What happened? (probe for both helpful and unhelpful responses)?

- And talking to the GP receptionist when you booked, how was that?

(if there was a Long Covid diagnosis) How was your diagnosis of Long Covid communicated to you?

- Talk us through what the conversation was like?
- How did you feel about that?
- What can you remember about the types of things your GP said?

How often do you talk to your GP since your diagnosis?

- Do you speak to the same GP?
- If it varies: how come?

Has there been any changes to the discussion of long Covid with your GP since diagnosis?

- Talk us through the changes?

What if anything do you talk to your GP about in terms of your family situation and the impact of long Covid?

Have you had any referral or any treatment/advice offered for long Covid?

- How were they offered?
  If dietary supplements, rest or graded exercise or PACE (progressive accelerating cardiopulmonary exertion) are mentioned; please ask how these were explained and how the conversation went.

What, if any, concerns about Long Covid did you talk to your GP about?

- How were these concerns responded to?

Is there anything your GP said that was helpful/unhelpful?

We will be using these interviews to develop training for GPs about how to talk to patients with LC? DO you have any recommendations for this training, based on what has happened to you?

Have you been referred to or referred yourself to any services specially designed for people with Long Covid? *Tell us all about it! When, how often, what happened, any surprises, when you are going back etc etc*

Self-management for LC

Have you taken any medicine, over the counter remedies or anything else to manage your LC symptoms, either ones prescribed for you or ones you have bought/used yourself (*might include pain killers, other over-the-counter medications for fever/pain control)?*

Have you taken any nutritional supplements, vitamins, particular foods or changed your diet at all in relation to Long Covid? What (why, who) prompted you to make this change? Has it helped?

Have you used any complementary / alternative approaches (what, why, attitude to)?

Recovery

Would you say that you have 'recovered' from having Long Covid or that you feel like you're in the process of recovering? If 'recovered'/recovering

- When (how long after first symptoms) did you recognise or feel confident that you were getting better? Did some aspects of your health improve quicker? Did some improve more slowly?
- Were any aspects of your process of recovering from Long Covid particularly encouraging or frustrating?
- What do you think has helped you to recover?
- Were there any things that you think slowed your recovery?
- Is there any advice that you think could be helpful in speeding or supporting the recovery of others with Long Covid?

Do you feel protected from catching COVID-19 again?

Have you had a COVID-19 vaccination?
(*If had vaccination)* How was your experience of vaccination (which vaccine did you have? When? Did you experience side effects? Did you notice any change in your LC symptoms after vaccination?)

Closing questions/reflections

Service improvement

There is still a lot that is unknown about LC – what are the questions that you would like to see answered in the future?

Do you have any messages for others affected by the condition (including advice on what they might talk to their doctors about)

(To ask those who had some interaction with some part of the health service):
People who have been through treatment in the NHS often notice things that don't seem to make sense, or seem inefficient or confusing. Sometimes they have really good ideas about

what might improve things. Is there anything you noticed that you think could/ should be changed to improve how people with Long Covid are treated and supported to recover?

What services aren't provided that you'd like to see?
Could services be better organised?
Do you think there are some services you don't need or services which could be replaced with something else?
What's the one change that you would most like to see? If you were asked to say what good care for someone with your condition looks like, what would you say?
Or what are the features of really good care for someone with Long Covid?

Is there anything else you would like to talk about?

Interview Guide for Experiences of Long Covid in Families study – parents or young people (carers of someone with Long Covid)

NB. Participants may occupy dual roles and be both carers and patients. Topic guide can be modified accordingly.

PREAMBLE for parents (who are caring for a child with Long Covid)
PART ONE: INVITATION TO TELL THEIR OWN STORY OF LONG COVID IN THE FAMILY

In this interview, we would like to hear about your experiences of how Long Covid has affected you and your family. I am interested in whatever you would like to tell me about your experiences but please do feel free to take a break whenever you need to – we can continue another day if you get tired.

In the first part of the interview I will ask you to tell me about your family's and your child's experiences.
After you have finished your story, I will ask you a few extra questions if that's OK.

PART TWO: FOLLOW-UP PROMPTS TO COVER ANY TOPICS NOT COVERED IN INTERVIEWEE'S NARRATIVE - PARENTS AND YOUNG PEOPLE CARING FOR A FAMILY MEMBER

Initial awareness of Long Covid and ideas about own/ family vulnerability
The purpose of this area of questioning is to develop an understanding of how Long Covid became a concern for the participant and their family and if they made any changes in response (beyond those already in place around Covid)

First awareness of Long Covid

When did you first hear about Long Covid? Can you remember how you heard about it and what you thought it meant? Where did your information come from?

Did you have particular worries or concerns about yourself or members of your family and those close to you?

For example, did you hear anything in the media about some groups of people being more affected by Long Covid than others? How did you feel about that?

Did the awareness of Long Covid lead you to you make any additional changes (i.e. changes beyond those you might have already made in relation to COVID-19) to your daily life or to the people you would see?

Did other people in your family make changes as well? How did that work?

Experiences of family member becoming ill with Long Covid

Suspecting Long Covid

When did [family member] realise that they might have Long Covid? *(NB use terminology that participant has used to this point) OR*
When did you realise that [family member] might have Long Covid?
What was your/their life like at that time? (school life, work life, social life, family life)

Which month and year was it when they had COVID-19, and then when did you/they realise it was Long Covid?

If diagnosed: How did you feel about [family member] getting diagnosed with Long Covid? How did they feel about it? Has this changed over time?

Illness experience

(How) has [family member's] experience of having Long Covid changed over time? (Prompts: *How) have the symptoms changed over time? Are there any triggers that make their symptoms worse? Is there anything that helps with their symptoms? What have you/they tried? What happened when you/they tried this? What has been suggested by others? Are there any symptoms that particularly worry you or them – either now or thinking about the longer term?*)

(*If relevant – may have been previously fit and well*) Thinking about the health problems or any health conditions [family member] had before they developed Long COVID, have any of these have changed (got worse/better)

Who did you tell when you first had/thought [family member] had Long Covid? (did you tell people you have contact with regularly? What were their responses?)

Actions taken

Has anyone else helped care for/look after [family member] with you?

How did you do to manage in your day-to-day life when [family member] developed Long Covid? Did you make any changes to your usual routines? Did any other family members have to make changes to their daily life?

Were there important things that you weren't able to do while you were looking after [family member] (work, other caring responsibilities, religious activities)? How did you manage those things?

Family life

How has [family member's] long covid affected your day-to-day life? (*prompts: work, relationships, ability to do things you previously enjoyed, sleep etc*)

Can you tell me about how Long Covid has affected your family life?

Have there been any disruptions to key tasks in your family life e.g. to childcare, household routines, work, shopping, family-owned or run businesses etc.

If split or multiple households: can you tell me about how you've managed across households? How about with extended family?

Have there been any changes in your family roles? (*prompts: who works, childcare, household tasks)*

What was your day like yesterday? OR Talk me through what a typical day looks like for your family. Have there been times when it was more difficult? What has made it easier? Have you had to deal with anything similar to this before as a family? *If yes:* can you tell me about your earlier experiences?

If relevant: Do you have any concerns about regaining your independence / regaining normality?

Is there anything you continue to struggle with as a family? How does this make you feel about the future?

Are there any other ways in which having Long Covid is still affecting your day-to-day family life or your plans for the future? (use prompts as necessary)

- Impact on work school and other daily activities
- Impact on family and social life
- Returning to work/getting a new job (support from employer/colleagues, Occupational Health, adjustments, changed hours and positive and negative experiences)
- Financial issues (were you working, furloughed, did you get sick leave, did you get sick pay, state benefits, travel costs, insurances, change in outgoings,

for younger people: statement of special educational needs, disabled students allowance)
    - Things you've had to cancel, abandon or postpone

Was there anything you felt was particularly unfair about your family's experience of Long Covid?

<u>Information and care-seeking</u>

How was the communication with your GP? What went well? What didn't go well? Talk me through what the conversation was like.

Can we talk a bit more about info about LC?
What are the main sources of information that you have accessed or made use of in relation to Long Covid? What if anything has been helpful/unhelpful about this information?

To what extent do you think Long Covid is well understood? (by those around you, healthcare staff, employers)

Accessing healthcare for LC

How did you/[family member] come to understand that they have LC?
Has [family member] seen their GP about their LC? Did you go with them?

Can you talk me through how [family member] has found accessing healthcare for LC? *OR*

Can you talk me through how you've found accessing care for [family member's] LC?

What happened? (probe for both helpful and unhelpful responses)?

- And talking to the GP receptionist when you/they booked, how was that?

(if there was a Long Covid diagnosis) How was the diagnosis of Long Covid communicated to you?

- Talk us through what the conversation was like?
- How did you feel about that?
- What can you remember about the types of things the GP said?

How often do you talk to the GP since the diagnosis?

- Do you speak to the same GP?
- If it varies: how come?

Has there been any changes to the discussion of long Covid with the GP since diagnosis?

- Talk us through the changes?

What if anything do you talk to the GP about in terms of your family situation and the impact of long Covid?

Has [family member] had any referral or any treatment/advice offered for long Covid?

- How were they offered?
  If dietary supplements, rest or graded exercise or PACE (progressive accelerating cardiopulmonary exertion) are mentioned; please ask how these were explained and how the conversation went.

What, if any, concerns about Long Covid did you talk to the GP about?

- How were these concerns responded to?

Is there anything the GP said that was helpful/unhelpful?

We will be using these interviews to develop training for GPs about how to talk to patients with LC? DO you have any recommendations for this training, based on what has happened to your family?

Has [family member] been referred to or referred themselves to any services specially designed for people with Long Covid? *Tell us all about it! When, how often, what happened, any surprises, when you are going back etc etc*

Self-management for LC

Has [family member] taken any medicine, over the counter remedies or anything else to manage their LC symptoms, either ones prescribed for them or ones you/they have bought/used yourself (*might include pain killers, other over-the-counter medications for fever/pain control)?*

Have they taken any nutritional supplements, vitamins, particular foods or changed their diet at all in relation to Long Covid? What (why, who) prompted you/them to make this change? Has it helped?

Have you/they used any complementary / alternative approaches (what, why, attitude to)?

Recovery

Would you say that [family member] has 'recovered' from having Long Covid or that you feel like they're in the process of recovering? If 'recovered'/recovering

- When (how long after first symptoms) did you recognise or feel confident that they were getting better? Did some aspects of their health improve quicker? Did some improve more slowly?
- Were any aspects of the process of recovering from Long Covid particularly encouraging or frustrating?
- What do you think has helped them to recover?
- Were there any things that you think slowed the recovery?
- Is there any advice that you think could be helpful in speeding or supporting the recovery of others with Long Covid?

Do you feel that [family member] is protected from catching COVID-19 again?

If relevant: Has [family member] had a COVID-19 vaccination?

(*If had vaccination)* How was you're the experience of vaccination (which vaccine did they have? When? Did they experience side effects? Did you notice any change in you're their LC symptoms after vaccination?

Closing questions/reflections

Service improvement

There is still a lot that is unknown about LC – what are the questions that you would like to see answered in the future?

Do you have any messages for others affected by the condition (including advice on what they might talk to their doctors about)

(To ask those who had some interaction with some part of the health service):
People who have been through treatment in the NHS often notice things that don't seem to make sense, or seem inefficient or confusing. Sometimes they have really good ideas about what might improve things. Is there anything you noticed that you think could/ should be changed to improve how people with Long Covid are treated and supported to recover?

What services aren't provided that you'd like to see?
Could services be better organised?
Do you think there are some services you don't need or services which could be replaced with something else?
What's the one change that you would most like to see? If you were asked to say what good care for someone with your condition looks like, what would you say?
Or what are the features of really good care for someone with Long Covid?

Is there anything else you would like to talk about?
