# Supplementary material for: Impact of Long Covid on the school experiences of children and young people: a qualitative study
Source: BMJ Open. 2023 Sep 19;13(9):e075756. doi: 10.1136/bmjopen-2023-075756 (PMC10510910; doi:10.1136/bmjopen-2023-075756)
Supplement: Author's manuscript [file bmjopen-2023-075756.draft_revisions.pdf]

BMJ Open is committed to open peer review. As part of this commitment we make the peer review history of every article we publish publicly available.

When an article is published we post the peer reviewers' comments and the authors' responses online. We also post the versions of the paper that were used during peer review. These are the versions that the peer review comments apply to.

The versions of the paper that follow are the versions that were submitted during the peer review process. They are not the versions of record or the final published versions. They should not be cited or distributed as the published version of this manuscript.

BMJ Open is an open access journal and the full, final, typeset and author-corrected version of record of the manuscript is available on our site with no access controls, subscription charges or pay-per-view fees (http://bmjopen.bmj.com).

If you have any questions on BMJ Open's open peer review process please email info.bmjopen@bmj.com

BMJ Open

The impact of Long Covid on the school experiences of children and young people: a qualitative study.

Journal:	*BMJ Open*
Manuscript ID	bmjopen-2023-075756
Article Type:	Original research
Date Submitted by the Author:	19-May-2023

Complete List of Authors:	MacLean, Alice; University of Stirling, Institute for Social Marketing and Health<br>Wild, Cervantee; University of Oxford, Nuffield Department of Primary Care Health Sciences<br>Hunt, Kate; University of Stirling, Institute for Social Marketing<br>Nettleton, Sarah; University of York, Sociology<br>Skea, Zoë; University of Aberdeen, Health Services Research Unit<br>Ziebland, Sue; University of Oxford, NDPCHS

Keywords:	COVID-19, Schools, QUALITATIVE RESEARCH, Adolescent

SCHOLARONE™
Manuscripts

I, the Submitting Author has the right to grant and does grant on behalf of all authors of the Work (as defined in the below author licence), an exclusive licence and/or a non-exclusive licence for contributions from authors who are: i) UK Crown employees; ii) where BMJ has agreed a CC-BY licence shall apply, and/or iii) in accordance with the terms applicable for US Federal Government officers or employees acting as part of their official duties; on a worldwide, perpetual, irrevocable, royalty-free basis to BMJ Publishing Group Ltd ("BMJ") its licensees and where the relevant Journal is co-owned by BMJ to the co-owners of the Journal, to publish the Work in this journal and any other BMJ products and to exploit all rights, as set out in our licence.

The Submitting Author accepts and understands that any supply made under these terms is made by BMJ to the Submitting Author unless you are acting as an employee on behalf of your employer or a postgraduate student of an affiliated institution which is paying any applicable article publishing charge ("APC") for Open Access articles. Where the Submitting Author wishes to make the Work available on an Open Access basis (and intends to pay the relevant APC), the terms of reuse of such Open Access shall be governed by a Creative Commons licence – details of these licences and which Creative Commons licence will apply to this Work are set out in our licence referred to above.

Other than as permitted in any relevant BMJ Author's Self Archiving Policies, I confirm this Work has not been accepted for publication elsewhere, is not being considered for publication elsewhere and does not duplicate material already published. I confirm all authors consent to publication of this Work and authorise the granting of this licence.

For peer review only

Title:
The impact of Long Covid on the school experiences of children and young people: a qualitative study.

Author names:

Alice MacLean[1], Cervantee Wild[2], Kate Hunt[1], Sarah Nettleton[3], Zoe Skea[4] and Sue Ziebland[2]

Full institutional mailing addresses:

[1]Institute for Social Marketing and Health, University of Stirling, Stirling, Scotland, FK9 4LA, UK
[2]Health Experiences Research Group, Nuffield Department of Primary Care Health Sciences, University of Oxford, OX2 6GG, UK
[3]Department of Sociology, University of York, Heslington East Campus, York, England, YO10 5GD, UK
[4] Health Service Research Unit, University of Aberdeen, Aberdeen, Scotland, AB25 2DZ, UK

Email addresses:

alice.maclean@stir.ac.uk
cervantee.wild@phc.ox.ac.uk
kate.hunt@stir.ac.uk
sarah.nettleton@york.ac.uk
z.skea@abdn.ac.uk
sue.ziebland@phc.ox.ac.uk

For peer review only

Abstract

Objectives To explore the impact of Long Covid on the school experiences of children and young people (CYP).

Design Qualitative study using narrative interviews.

Participants 22 CYP (aged 10-18 years, 15 female) with Long Covid and 15 parents/caregivers (13 female) of CYP (aged 5-18 years) with Long Covid.

Setting Interviews were conducted between October 2021 and July 2022 via online video call or telephone. Recruitment routes included social media, LC support groups, clinicians, community groups and snowballing.

Results Three key findings were identified. Finding 1: Going to school is a valued part of CYP's lives and participants viewed educational attainment as important for their future trajectories. Returning to school full-time was highlighted as a key part of regaining 'normal life'. Finding 2: Attending school (in-person or online) with Long Covid is extremely difficult; even a gradual return required CYP to balance the impact of being at and engaging with school, with the need to manage symptoms to prevent relapse. Often this meant prioritising school and rest over other aspects of their lives. Finding 3: School responses to CYP with Long Covid were reported to be mixed and hampered by difficulties communicating with healthcare professionals during the pandemic and a lack of awareness of Long Covid among healthcare and education professionals. Participants viewed supportive school responses as staff believing, understanding and taking them seriously, alongside schools offering tailored and flexible adaptations which allowed engagement with school while limiting any deterioration of symptoms.

Conclusions This study describes how Long Covid affects the school experiences of CYP and generates recommendations for supportive school responses alongside supportive healthcare professionals. Further research could explore the approaches that facilitate a successful return to school for CYP with Long Covid and investigate education professionals' perspectives on support they require to positively engage with returning pupils.

Strengths and limitations of this study

- To our knowledge, this is the first qualitative study to explore the impact of LC on CYP's experiences of school.
- This study utilises rigorous methods and draws on rich accounts from a diverse sample of CYP with LC, and parents of CYP with Long Covid, across the UK.
- A limitation of the study is that it does not include the perspectives of education professionals on their experiences of engaging with CYP with LC and the support they need to do this.
- The composition of our sample prevented a systematic comparison of accounts by age, gender, ethnicity or social class, thus preventing investigation of whether CYP with LC experience similar expectations around responding to illness with stoicism, control and independence (especially while at school) which previous research has highlighted.

For peer review only

INTRODUCTION

Children and young people (CYP) infected with SARS-CoV-2 are at lower risk of hospitalisation and mortality compared to adults[1] but, like adults, can experience symptoms which persist long after initial infection[2-4]. These ongoing symptoms are variously described as post-COVID-19 syndrome, post-COVID-19 condition and post-acute sequelae of SARS-CoV-2 (PASC) or Long Covid (LC), the name first used by adults with persisting symptoms[5]. There is an increasing research focus on LC in adults (e.g. [6-9]), but limited high-quality evidence on CYP and ongoing uncertainties, including the prevalence, risk factors, mechanisms and effective management of LC in CYP[10-13]. Here, we describe accounts from CYP and parents of the impacts of LC on education and school-related challenges and recommend ways that schools could support CYP with LC.

Long Covid has been described as a "frequent legacy of acute SARS-CoV-2 infection, affecting over 10% of patients" [7] (p.1891) with wide-ranging symptoms. Its clinical case definition in adults describes: "a history of probable or confirmed SARS-CoV-2 infection, usually 3 months from the onset of Covid-19 with symptoms…that last for at least 2 months and cannot be explained by an alternative diagnosis"[14] and research suggests CYP have comparable symptom experiences to adults[15]. Children and adults report: many persistent symptoms affecting multiple body systems[11], with fatigue, headache, cognitive impairment and shortness of breath being common[2 16-18]; and symptoms that fluctuate in severity[11], with new symptoms emerging potentially months after initial infection. A higher prevalence of persistent symptoms has been reported in females[13 19]. Accurately measuring symptoms of Long Covid over time is challenging given the emergence of new variants of Covid-19, the potential impact of vaccines and the difficulty of ruling out the influence of other viral infections[3 20].

Research into the social and academic impacts of LC on CYP is lacking and existing evidence is mixed. A Spanish study followed (for five months) 50 CYP with LC (defined as ongoing symptoms for 12+ weeks) and reported that 18% were unable to attend school, 34% had a reduced school schedule, 66% showed decreased school performance and 68% had stopped extracurricular activities[15]. Systematic reviews have highlighted heterogeneous findings relating to the impact of LC on CYP's lives. Pellegrino et al[12] included five studies which reported limitation in daily function affecting school attendance (in 10.5 to 58.9% of participants across the studies). Franco et al[21] reviewed 25 studies which included wellbeing and recovery outcomes in CYP, five of which reported outcomes relating to 'changes in work/occupation and study (school attendance)'. The authors suggested "most children with persistent symptoms reported no substantial impairment in their school functioning at 3–6-month follow-ups" (p.12). However, it has been noted that the evidence in CYP is limited, heterogeneous and largely based on low-quality studies[12].

We found no qualitative studies on the impacts of LC on CYP's school experiences. However, research with CYP with chronic fatigue syndrome (CFS)/myalgic encephalomyelitis (ME), a condition with some similarities to LC[22 23], highlights detrimental impacts on social, emotional and academic aspects of schooling. For CYP with CFS/ME, disrupted schooling "has a significant impact on the self [resulting] in a shift from a perceived normal trajectory of academic achievement and independence to one that is uncertain"[24] (p.10), and some educational support and adaptations underpinned by evidence-based awareness-raising in schools have been recommended[25-28].

METHODS

This paper draws on narrative interviews undertaken to improve understanding of the experience of LC in households with CYP from diverse backgrounds. In this analysis we draw on interviews with CYP (aged

For peer review only - http://bmjopen.bmj.com/site/about/guidelines.xhtml

10-18 years) with LC and parents/caregivers of CYP (aged 5-18 years) with LC, some of whom had LC themselves.

Patient and public involvement

An advisory panel, including patient and public involvement representatives with lived experience of LC or of caring for a child or young person with LC, had input into all aspects of the study conduct, including content of the interview topic guide and recruitment methods.

Recruitment and sampling

Recruitment routes included social media, LC support groups, clinicians, community groups and snowballing. We aimed for maximum variation sampling[29], to capture diversity by age, gender, ethnicity, geographical location, and social class. Potential participants were eligible if they, or the person they cared for, had self-identified ongoing symptoms 12+ weeks after initial Covid-19 infection. CYP were eligible if they were 10 years or over. Parents were eligible if their child with LC was five years or over. Age-appropriate information sheets were provided to potential participants.

Data collection

Narrative interviews (n=37) were conducted (between October 2021 and July 2022) via online video call or telephone, and were video and/or audio recorded, depending on participant preference. Verbal consent was recorded at the start of their interview. Those under 16 years gave assent and their parent/caregiver gave proxy consent. Participants were offered a £30 voucher to thank them for sharing their time and experiences.

Interviews began with an open narrative where participants were asked to recount events since they/their child first experienced signs of Covid. The second part used topic guides (different for CYP and parent interviews) with prompts, including questions about how Long Covid had affected school. Interviews typically lasted between 25 and 90 minutes, with some conducted over multiple shorter sessions to accommodate participant fatigue or other symptoms.

Data analysis

Interviews were transcribed verbatim, checked for accuracy and imported into NVivo (March 2020 version) to aid organisation and coding of data. We used thematic analysis[30] to inductively code the data. After initial familiarisation with transcripts, we developed a coding framework of broad themes, which was refined throughout the coding process. Three researchers coded the transcripts (CW, ZS, SN). All data coded to the broad 'school/education' theme were then further analysed (by AM) using the mind-mapping 'one sheet of paper' (OSOP) technique[31]. This process generated three sub-themes, as described below with interview extracts (IE) to illustrate the range of views (longer extracts are presented in boxes 1-3). All names are pseudonyms. When quoting a parent who spoke in their child's interview, we use the convention 'mother of Gemma (16, LC 19 months)'.

Ethics

Ethical approval was granted by Berkshire Ethics Committee (12/SC/0495).

RESULTS

Participants

The 37 participants included in this analysis comprised of n=8 children aged 10-12 years (n=4 female), n=14 young people aged 13-18 years (n=11 female) and n=15 parents of a child/young person with LC

For peer review only

(n=13 female), five of whom also had LC themselves. Table 1 displays participant characteristics of the CYP and parents.

Children						
**Pseudonym**	**Age**	**Gender**	**Ethnicity**	**Month of infection**	**Month of interview**	**Time affected by symptoms (months)**
Kieran	12	Male	White British	Mar-20	Dec-21	19
Shay	12	Male	White British	Mar-20	Dec-21	19
Mae	11	Female	White British	Jul-20	Mar-21	8
Josie	10	Female	Mixed race	Jan-22	Apr-22	3
Xavier	12	Male	Slovak/Algerian	Sep-21	May-22	8
Amelia	12	Female	White British	May-20	Jun-22	25
Rohaan	12	Male	British Pakistani	Jan-22	Jun-22	6
Noor	11	Female	British Pakistani	Sep-21	Jul-22	10
**Young People**						
Matt	16	Male	White British	Nov-20	Nov-21	12
Molly	16	Female	White Northern Irish	Sep-21	Dec-21	3
Fred	14	Male	White British	Dec-20	Mar-22	15
Holly	14	Female	White British	Sep-21	Mar-22	6
Erin	15	Female	White British	Sep-21	Mar-22	6
Gemma	16	Female	White British	Aug-20	Mar-22	19
Alana	13	Female	White British	Jul-21	Apr-22	9
Faye	14	Female	White British	Oct-20	Mar-22	17
Rory	13	Male	White British	Sep-21	Apr-22	7
Hala	15	Female	British Pakistani	Jul-21	Jun-22	11
Layla	14	Female	White British	Nov-21	June-22	7
Dina	16	Female	British Pakistani	Sep-21	June-22	9
Raabia	17	Female	British Pakistani	Sep-21	June-22	9
Inaya	13	Female	British Pakistani	Jan-22	Jun-22	6
**Parents**						
**Pseudonym**	**Age of child with Long Covid**	**Gender**	**Ethnicity**	**Month of child's infection**	**Month of interview**	**Time child affected by symptoms (months)**
Caitlin	6	Female	Ashkenazi Jewish	Mar-20	Nov-21	18
Marissa	16	Female	White British	Nov-20	Nov-21	12
Izzy	12	Female	White British	Mar-20	Dec-21	19
Martin	16	Male	Northern Irish	Sep-21	Dec-21	3

Table 1: Participant characteristics

For peer review only

Ross	13	Male	White British	Mar-20	Dec-21	19
Laura	6	Female	White British	May-21	Mar-22	10
Angela	8	Female	White British	Nov-21	Apr-22	5
Jan	10	Female	White British	Jan-22	Apr-22	3
Evelynn	8	Female	White British Manx	Mar-21	Apr-22	13
Lorna	10	Female	White British	Oct-21	Apr-22	6
Olivia	11	Female	White British	Oct-21	Apr-21	6
Freja	12	Female	Slovak	Sep-21	May-22	8
Haniya	13	Female	British Pakistani	Nov-21	Jun-22	7
Chrissie	6	Female	Black Caribbean	Feb-22	Jun-22	4

In interviews with CYP, some parents chose to be present, either sitting beside their child or elsewhere in the room while the interview was conducted. When parents were present, some spoke very little in the interview and others contributed significantly. The sample included four families where more than one member took part individually, and one family contributed three separate interviews (two with children with LC and one with their parent).

Analysis identified three key themes: CYP's desire to get back to school; their experiences of being in school whilst still affected by Long Covid; and schools' responses to their illness.

"I just want to be normal again": education as a normal and valued aspect of life for CYP
Most CYP's accounts indicated that they had been too ill to attend school regularly or undertake online learning and some were not attending school at all when interviewed. GP and hospital appointments also caused frequent school absences. Analysis revealed a strong desire for their lives to return to the way they were before having Covid and CYP spoke about being able to go back to school as a major means of regaining some normality (see Box 1-IE 1&2). Children's eagerness to return to school was also evident in parents' accounts (see Box 1-IE 3).

Expressing their desire to return to school ("*I would give so much to go back to school full-time. I miss it a lot*" (Faye, 14, LC 17 months)) contradicts common stereotyping of children, and particularly teenagers, as lazy or reluctant to engage with school. In contrast, CYP's accounts portrayed school absence as making them stand out from their peers, going against the 'normality' of full-time school for people their age. Frequent or extended absences were described as stressful and isolating, leading to feelings of being left behind academically and socially (see Box 1-IE 4). The unpredictable and variable nature of symptoms was particularly distressing because CYP did not know how long disruption to their schooling would last ("*It's never ending […] you're in a maze and you turn around the corner and 'Oh, this is going to be the end,' but it's a dead end, that's what it feels like*" (Fred, 14, LC 15 months)).

"School is still like a lot of energy": experiences of returning to school with LC
For many CYP we talked to, going back to school had not represented the hoped for 'return to normal' and they commonly highlighted extreme fatigue ("*after school, I would come home [and] sleep for ages [and] I've never done that before*" (Faye, 14, LC 17 months); "*I couldn't really do anything [with friends] at break. I was just resting. I struggled going up the stairs. I can't do PE. Yeah, I just feel tired after every*

For peer review only

lesson” (Rory, 13, LC 7 months)). Attempts to return to school invariably led to “*crashes*”, “*huge relapses*” or feeling “*exhausted*”, followed by needing more time off to recover (see Box 2-IE 1).

Navigating school buildings was difficult and made symptoms worse (“*school is still a lot of energy because I go to a very big school […] so that's a lot of walking about*” (Alana, 13, LC 9 months); “*I've got third floor, second floor, first floor, the ground floor, I have to go up and down stuff [and] I get tired*” (Rohaan, 12, LC 6 months); *“[My classroom] used to be upstairs and that was the hardest bit because before I stopped walking [and got a wheelchair], I had to go upstairs and my knees were so sore*” (Josie, 10, LC 3 months)). As well as fatigue, other physical symptoms impacted their ability to learn (see Box 2-IE 2&3).

Many highlighted difficulties caused by cognitive impairment (“*I can't concentrate with reading*” (Shay, 12, LC 19 months); “*I sometimes get brain fog, I'm sometimes just lost*” (Fred, 14, LC 15 months)). Those who previously enjoyed school described this as particularly distressing (see Box 2-IE 4). For many, learning online from home was not an appropriate solution. Gemma (16, LC 19 months) who was no longer going to school when interviewed, said “*[for two months] I was trying to do a full week at school from home while not feeling well and I just couldn't cope with it, so I was removed from all my classes*”. Those with brain fog and fatigue struggled to follow online learning (see Box 2-IE 5). There were also social and emotional difficulties involved in returning to school. Some found it distressing and isolating to feel they were falling behind their peers academically, and some described how their absences from school contributed to the disintegration of friendships (see Box 2-IE 6&7).

CYP who were able to attend school, at least part-time, struggled to make a successful return while managing their symptoms. Secondary school pupils spoke about being aware of the importance of education for their future and wanting to do well in exams to keep further education and career options open (“*I want to get good GCSEs*” (Layla, 14, LC 7 months)). Balancing the impact of working hard at school with managing their symptoms and preventing relapses was difficult. Sacrifices and prioritisation were often recounted, such as concentrating on fewer school subjects (see Box 2-IE 8) or restricting social activities to conserve energy for going to school (“*I'm not doing anything outside of school really […] I go to school, I go to sleep, […] which is difficult*” (Erin, 15, LC 6 months)).

“It's a mixed bag”: Schools' responses to CYP with LC

Parents spoke about alerting their child's school to the health challenges they were experiencing and explaining their absences. This could be difficult due to the varied and unpredictable nature of symptoms (“*I said to [son's school], 'No, he doesn't have fever, he is just exhausted, I can't send him to school, I'm sorry,' so of course [the school is] concerned*” (Freja, parent of 12-year-old, LC 8 months)). One parent, whose child caught Covid in November 2021, said she had tried to explain LC to her daughter's school “*because it's new to them as well*” (Angela, parent of 8-year-old, LC for 5 months). Parents understood schools require explanations for absences, ideally “backed-up” by validation from healthcare professionals. However, the pandemic had made it harder to facilitate communication between healthcare professionals and schools.

When Covid cases were high in school, some parents preferred their child to stay away to avoid reinfection (“*cases are so high [that] I've got no interest in sending [my children to school]*” (Izzy, parent of 12-year-old, LC 19 months); *“[son] went back [to school] again [and] then we had to miss the last couple of days [before Christmas holidays] because cases were going through the roof*” (Ross, parent of 13-year-old, LC 19 months)). A paediatrician's reported advice that school was “*absolutely the best place*” for her daughter, was unwelcome to a parent who felt her concerns about reinfection and its impacts were dismissed (“*I kept saying, 'But what happens if she goes back to school and gets Covid [again] on top of*

For peer review only

how she's feeling?'" (mother of Gemma, 16, LC 19 months)). Another mother, whose daughter caught Covid in May 2021, said school had ignored her fears about the risk of reinfection and she felt they were applying pressure to send her daughter back to school (see Box 3-IE 1).

A perceived lack of integration of care across health and education settings was compounded by the fact that LC in CYP was a new condition that was neither widely recognised nor well understood (see Box 3-IE2). Even when contact was made between health and education practitioners, it was then hard for parents and CYP to plan how much and how often they could attend school because symptoms were unpredictable, varying day-to-day or week-to-week and activity needed to be balanced against potential relapses.

Other participants recounted positive experiences once a link had been made between school and a healthcare professional who suggested ways to manage symptoms at school (e.g., attending part-time, not doing physical education, frequent rest breaks). Some schools were described as responsive, assertive, supportive, and flexible, by putting various adjustments in place (see Box 3-IE 3&4). Parents and CYP mentioned understanding and supportive teachers ("*my art teacher has been really good [and] worked out what work absolutely needed to be done [and] came up with the idea of basing my whole art project around Long Covid*" (Molly, 16, LC 3 months); *"[the teacher's] wife had chronic fatigue syndrome so he's really understanding and he's amazing with [my daughter]"* (Olivia, parent of 11-year old, LC 6 months)). CYP and parents wanted to feel that school staff believed them and took the impact of LC seriously *("[her teachers] are really flexible if she needed a break, she can go and sit down: there's no questions asked*" (Evelynn, parent of 8-year-old child, LC 13 months)).

However, a few parents and CYP said that schools had not implemented systems to facilitate reintegration ("*I had to tell [my teachers about Long Covid] myself [because] all school told them was that I was going part-time*" (Faye, 14, LC 17 months); *"[during PE] they forgot I couldn't do stuff [and] I just had to sort of watch them do fun stuff*" (Rory, 13, LC 7 months)). Others, including some interviewed in 2022, described a lack of awareness and understanding of LC among school staff. Fred (14, LC 15 months), interviewed in March 2022, described his teachers' responses as a "*mixed bag*"; some understanding teachers said, "*don't push yourse*lf" while others were "*the opposite and want me to do the same things and the same tests [as other pupils]*". Even when healthcare professionals had written to the school, there were still accounts of disbelief, a lack of understanding and even threats of fines for non-attendance (see Box 3-5).

DISCUSSION

Our findings highlight the importance of school to CYP with LC and how returning to school was central to CYP's much hoped for 'return to normal'. CYP described absences from school as stressful and isolating and placed high importance on returning to school full-time. However, re-engaging with school could lead to relapses, and further absences, meaning CYP had to learn how to deal with the demands of school without pushing themselves to "crashing" point. Often this meant prioritising school and rest over all other aspects of life. The various school responses to LC that participants described have highlighted the importance of validation of CYP's experience of LC by healthcare professionals and informed recommendations for supporting CYP with LC at school to minimise adverse educational, social and mental health sequelae of having LC in childhood and adolescence (see Box 4).

To our knowledge, this is the first qualitative study to explore the impact of LC on CYP's experiences of school. A further strength of our study is that it utilises rigorous methods and draws on rich accounts from a diverse sample of CYP with LC across the UK. A limitation is that it does not include the perspectives of education professionals on their experiences of engaging with CYP with LC and the support they need to

For peer review only

do this. The composition of our sample prevented a systematic comparison of accounts by age, gender, ethnicity or social class, thus preventing investigation of whether CYP with LC experience similar expectations around responding to illness with stoicism, control and independence (especially while at school) which previous research has highlighted[32]. It is also important to acknowledge that participants' accounts may have been impacted by the fact that they were being filmed/audio-recorded for a website. However, all participants were given the option of safeguarding their identity by making their data fully anonymous.

There are few existing studies with which to compare our findings, although there are some parallels with studies of the impact of CFS/ME and other long-term health conditions on school experiences, particularly the importance of school and its centrality to CYP's hopes to return to a 'normal' trajectory[24 27 33 34]. School absence because of LC marked CYP out from peers and they reported feeling stressed, isolated [35 36] and worried about falling behind academically and socially. These negative emotions were compounded by not knowing how long this 'break from normality' and uncertain trajectory would continue, as has been reported for CYP with CFS/ME, juvenile idiopathic arthritis and other long-term conditions[24 35 36]. Echoing the "ripple effect" of CFS/ME on CYP's social, emotional and academic functioning[25], our findings demonstrate the impact of LC on CYP's participation in many aspects of school life, highlighting their distress about their inability to fully reintegrate with peers in the way they had hoped. As CYP placed a high value on education and often prioritised school (and rest) over all other aspects of their lives, there is a need to find ways of helping CYP with LC and other long-term conditions to minimise school demands so that they can also participate in other aspects of their lives[28]. As reported for CYP with CFS/ME[27 28], we found that schools' responses were experienced as particularly supportive when participants felt believed and understood by school staff (often following validation of the CYP's condition by a healthcare professional) and when schools instigated tailored and flexible adaptations to help CYP engage with school while preventing relapse. The recommendations for healthcare and education professionals stemming from our findings are underpinned by values such as empathy, respect, openness and flexibility. They echo the "approaches of positive schools" and key actions in the role of schools and teachers in engaging young people with health conditions outlined by Hopkins et al[37] (p.32).

A notable difference from research on other illnesses is that LC emerged as a new condition during a global pandemic. There is an expanding literature on the processes that adults with LC have gone through to recognise their symptoms as legitimate and worthy of investigation, support and treatment[5 8 32]. However, little is known about equivalent experiences of CYP with LC and their parents/caregivers. Indeed, it has been suggested that resistance to recognising Long Covid as a clinical entity has been even more pronounced for CYP[18]. Research on paediatric CFS reports that CYP and parents still "often report feeling misunderstood and disbelieved by medical and educational services" despite CFS being a defined diagnostic entity since 1991[38] (p.12). Studies of CYP with CFS/ME highlight the importance of diagnosis and healthcare professional validation in legitimising the illness[27 28]. Our findings suggest that harnessing this "power of diagnosis" has been especially problematic for CYP with LC due to the novelty of LC and recency of its emergence within a time of unprecedented pressures on healthcare services, which limited parents' ability to facilitate links between healthcare and education professionals. While Simila et al[28] found that online teaching was perceived as helpful by CYP with CFS/ME during periods of lockdown, this was not always the case for CYP with LC in our study. Again, this highlights the need for further research on how educational adaptations can be tailored to the ways that LC symptoms impact upon CYP's abilities to learn to minimise adverse health, social and educational outcomes in later life. For some parents in our study, a further tension existed around whether school was a safe place for CYP with LC, particularly when high case numbers in schools fuelled concerns that reinfection with Covid-19 might exacerbate or prolong LC symptoms. This novel aspect of our findings also requires further exploration.

Our findings suggest ways that schools can support CYP with LC and highlight the need to raise awareness of LC among healthcare and education professionals. However, there is a need for further research on appropriate educational and social adaptations for CYP with LC, and increased knowledge and understanding of LC in schools and the most supportive ways that school can respond. Further research might explore experiences by age, gender, social class and ethnicity to identify where interventions could be focussed. Research with educational professionals could investigate how best to facilitate positive engagement with CYP with LC and their parents. Overall, it is important that CYP with LC are supported to engage with school in ways that facilitate recovery and minimise the impact of LC on their lives, physical and mental health and education longer term.

FUNDING STATEMENT
This work is independent research funded by the National Institute for Health and Care Research (NIHR) (COV-LT2-0005). The views expressed in this publication are those of the author(s) and not necessarily those of NIHR or The Department of Health and Social Care.

COMPETING INTERESTS
None declared.

CONTRIBUTORS
SZ and KH developed the study, with input from CW, SN, ZS, AM and the research advisory group which included CYP with LC and parents of CYP with LC. CW led the day-to-day management of the project, supported by SZ, KH, SN, AM and ZS. The qualitative interviews were conducted by CW, SN, KH, AM and ZS; all are highly experienced qualitative researchers. CW, ZS and SN coded the interview transcripts. AM further analysed all data relating to school/education using the mind-mapping 'one sheet of paper' (OSOP) technique. AM drafted the manuscript, and all authors provided critical comments on drafts, and read and approved the final manuscript. All authors contributed to revisions of the paper.

PATIENT CONSENT FORM
Not applicable.

DATA SHARING STATEMENT
The University of Oxford holds the copyright for the full interview transcripts and may grant data sharing permission upon request.

ACKNOWLEDGEMENTS
We would like to thank all the children, young people and parents who took part in interviews, especially as many of our participants were still very affected by their Long Covid symptoms and had limited physical and cognitive resources. We would like to thank the funders of our research and our colleagues at the Health Experiences Research Group in Oxford. Thank you especially to Dr Anna Dowrick for helpful comments on an earlier draft.

REFERENCES

1. Chua PEY, Shah SU, Gui H, et al. Epidemiological and clinical characteristics of non-severe and severe pediatric and adult COVID-19 patients across different geographical regions in the early phase of pandemic: a systematic review and meta-analysis of observational studies. *J Investig Med* 2021;69(7):1287-96. doi: 10.1136/jim-2021-001858 [published Online First: 20210616]

For peer review only

2. Behnood SA, Shafran R, Bennett SD, et al. Persistent symptoms following SARS-CoV-2 infection amongst children and young people: A meta-analysis of controlled and uncontrolled studies. *J Infect* 2022;84(2):158-70. doi: 10.1016/j.jinf.2021.11.011 [published Online First: 20211120]
3. Kikkenborg Berg S, Palm P. Playing catch with long COVID - understanding a new disease. *Lancet Reg Health Eur* 2023;25 doi: https://doi.org/10.1016/j.lanepe.2022.100568
4. Ludvigsson JF. Case report and systematic review suggest that children may experience similar long-term effects to adults after clinical COVID-19. *Acta Paediatr* 2021;110(3):914-21. doi: 10.1111/apa.15673 [published Online First: 20201203]
5. Callard F, Perego E. How and why patients made Long Covid. *Soc Sci Med* 2021;268:113426. doi: 10.1016/j.socscimed.2020.113426 [published Online First: 20201007]
6. Macpherson K, Cooper K, Harbour J, et al. Experiences of living with long COVID and of accessing healthcare services: a qualitative systematic review. *BMJ Open* 2022;12(1):e050979. doi: 10.1136/bmjopen-2021-050979 [published Online First: 20220111]
7. Mantovani A, Morrone MC, Patrono C, et al. Long Covid: where we stand and challenges ahead. *Cell Death Differ* 2022;29(10):1891-900. doi: 10.1038/s41418-022-01052-6 [published Online First: 20220907]
8. Rushforth A, Ladds E, Wieringa S, et al. Long Covid - The illness narratives. *Soc Sci Med* 2021;286:114326. doi: 10.1016/j.socscimed.2021.114326 [published Online First: 20210819]
9. Subramanian A, Nirantharakumar K, Hughes S, et al. Symptoms and risk factors for long COVID in non-hospitalized adults. *Nat Med* 2022;28(8):1706-14. doi: 10.1038/s41591-022-01909-w [published Online First: 20220725]
10. Franco JVA, Garegnani LI, Oltra GV, et al. Long-Term Health Symptoms and Sequelae Following SARS-CoV-2 Infection: An Evidence Map. *Interntional Journal of Environmental Research and Public Health* 2022(a);19 doi: https://doi.org/10.3390/ijerph19169915
11. Lopez-Leon S, Wegman-Ostrosky T, Ayuzo Del Valle NC, et al. Long-COVID in children and adolescents: a systematic review and meta-analyses. *Sci Rep* 2022;12(1):9950. doi: 10.1038/s41598-022-13495-5 [published Online First: 20220623]
12. Pellegrino R, Chiappini E, Licari A, et al. Prevalence and clinical presentation of long COVID in children: a systematic review. *Eur J Pediatr* 2022;181(12):3995-4009. doi: 10.1007/s00431-022-04600-x [published Online First: 20220915]
13. Stephenson T, Shafran R, Ladhani SN. Long COVID in children and adolescents. *Curr Opin Infect Dis* 2022;35(5):461-67. doi: 10.1097/QCO.0000000000000854 [published Online First: 20220803]
14. WHO. A clinical case definition of post COVID-19 condition by a Delphi consensus 2021 [updated 6 October 2021. Available from: https://www.who.int/publications/i/item/WHO-2019-nCoV-Post_COVID-19_condition-Clinical_case_definition-2021.1 accessed 16th November 2022.
15. Gonzalez-Aumatell A, Bovo MV, Carreras-Abad C, et al. Social, Academic, and Health Status Impact of Long COVID on Children and Young People: An Observational, Descriptive, and Longitudinal Cohort Study. *Children (Basel)* 2022;9(11) doi: 10.3390/children9111677 [published Online First: 20221031]
16. Aiyegbusi OL, Hughes SE, Turner G, et al. Symptoms, complications and management of long COVID: a review. *J R Soc Med* 2021;114(9):428-42. doi: 10.1177/01410768211032850 [published Online First: 20210715]
17. Chen C, Haupert SR, Zimmermann L, et al. Global Prevalence of Post-Coronavirus Disease 2019 (COVID-19) Condition or Long COVID: A Meta-Analysis and Systematic Review. *J Infect Dis* 2022;226(9):1593-607. doi: 10.1093/infdis/jiac136
18. Izquierdo-Pujol J, Moron-Lopez S, Dalmau J, et al. Post COVID-19 Condition in Children and Adolescents: An Emerging Problem. *Front Pediatr* 2022;10:894204. doi: 10.3389/fped.2022.894204 [published Online First: 20220511]

For peer review only

19. Kikkenborg Berg S, Palm P, Nygaard U, et al. Long COVID symptoms in SARS-CoV-2-positive children aged 0-14 years and matched controls in Denmark (LongCOVIDKidsDK): a national, cross-sectional study. *Lancet Child Adolesc Health* 2022;6(9):614-23. doi: 10.1016/S2352-4642(22)00154-7 [published Online First: 20220623]
20. Sakurada Y, Otsuka Y, Tokumasu K, et al. Trends in Long COVID Symptoms in Japanese Teenage Patients. *Medicina* 2023;59 doi: https://www.mdpi.com/1648-9144/59/2/261
21. Franco JVA, Garegnani LI, Oltra GV, et al. Short and Long-Term Wellbeing of Children following SARS-CoV-2 Infection: A Systematic Review. *Int J Environ Res Public Health* 2022(b);19(21) doi: 10.3390/ijerph192114392 [published Online First: 20221103]
22. Siberry VGR, Rowe PC. Pediatric Long Covid and Myalgic Encephalomylitis/Chronic Fatigue Syndrome. Overlaps and Opportunities. *The Pediatric Infectious Diseases Journal* 2022;41(4)
23. Wong TL, Weitzer DJ. Long COVID and Myalgic Encephalomyelitis/Chronic Fatigue Syndrome (ME/CFS)—A Systemic Review and Comparison of Clinical Presentation and Symptomatology. *Medicina* 2021;57
24. Parslow RM, Harris S, Broughton J, et al. Children's experiences of chronic fatigue syndrome/myalgic encephalomyelitis (CFS/ME): a systematic review and meta-ethnography of qualitative studies. *BMJ Open* 2017;7(1):e012633. doi: 10.1136/bmjopen-2016-012633 [published Online First: 20170113]
25. Brigden A, Shaw A, Anderson E, et al. Chronic fatigue syndrome/myalgic encephalomyelitis in children aged 5 to 11 years: A qualitative study. *Clin Child Psychol Psychiatry* 2021;26(1):18-32. doi: 10.1177/1359104520964528 [published Online First: 20201022]
26. Brigden A, Shaw A, Barnes R, et al. "The child's got a complete circle around him". The care of younger children (5-11 years) with CFS/ME. A qualitative study comparing families', teachers' and clinicians' perspectives'. *Health Soc Care Community* 2020;28(6):2179-89. doi: 10.1111/hsc.13029 [published Online First: 20200609]
27. Clery P, Linney C, Parslow R, et al. The importance of school in the management of Myalgic Encephalomyelitis/Chronic Fatigue Syndrome (ME/CFS): issues identified by adolescents and their families. *Health Soc Care Community* 2022;30(6):e5234-e44. doi: 10.1111/hsc.13942 [published Online First: 20220822]
28. Simila WA, Nost TH, Helland IB, et al. Factors related to educational adaptations and social life at school experienced by young people with CFS/ME: a qualitative study. *BMJ Open* 2021;11(11):e051094. doi: 10.1136/bmjopen-2021-051094 [published Online First: 20211118]
29. Coyne IT. Sampling in qualitative research. Purposeful and theoretical sampling; merging or clear boundaries? *J Adv Nurs* 1997;26(3):623-30. doi: 10.1046/j.1365-2648.1997.t01-25-00999.x
30. Braun V, Clarke V. One size fits all? What counts as quality practice in (reflexive) thematic analysis? *Qualitative Research in Psychology* 2021;18(3):328-52. doi: 10.1080/14780887.2020.1769238
31. Ziebland S, McPherson A. Making sense of qualitative data analysis: an introduction with illustrations from DIPEx (personal experiences of health and illness). *Med Educ* 2006;40(5):405-14. doi: 10.1111/j.1365-2929.2006.02467.x
32. Maclean A, Sweeting H, Hunt K. 'Rules' for boys, 'guidelines' for girls: Gender differences in symptom reporting during childhood and adolescence. *Soc Sci Med* 2010;70(4):597-604. doi: 10.1016/j.socscimed.2009.10.042 [published Online First: 20091122]
33. Harland MR, Parslow RM, Anderson N, et al. Paediatric chronic fatigue syndrome patients' and parents' perceptions of recovery. *BMJ Paediatr Open* 2019;3(1):e000525. doi: 10.1136/bmjpo-2019-000525 [published Online First: 20191202]
34. Parslow R, Patel A, Beasant L, et al. What matters to children with CFS/ME? A conceptual model as the first stage in developing a PROM. *Arch Dis Child* 2015;100(12):1141-7. doi: 10.1136/archdischild-2015-308831 [published Online First: 20151009]

For peer review only

35. Min M, Hancock DG, Aromataris E, et al. Experiences of living with juvenile idiopathic arthritis: a qualitative systematic review. *JBI Evid Synth* 2022;20(1):60-120. doi: 10.11124/JBIES-21-00139
36. Rasalingam A, Fegran L, Brekke I, et al. Young people with long-term health challenges experiences in transition to adulthood: A qualitative metasynthesis. *J Adv Nurs* 2021;77(2):595-607. doi: 10.1111/jan.14641 [published Online First: 20201127]
37. Hopkins L, Green J, Henry J, et al. Staying engaged: the role of teachers and schools in keeping young people with health conditions engaged in education. *Australian Journal of Educational Research* 2014:25-41.
38. Loades ME, Crawley E, Chalder T, et al. Paediatric chronic fatigue syndrome: 25 year perspective. *Clin Child Psychol Psychiatry* 2021;26(1):8-17. doi: 10.1177/1359104520978461 [published Online First: 20201210]

For peer review only

Box 1 "I just want to be normal again": education as a normal and valued aspect of life for CYP - illustrative interview extracts

IE 1. *"I just want to go back to school. Right, I know a lot of kids can't say that, but [I want to] just go back to my normal routine"* (Holly, 14, LC 6 months)

IE 2. *"I have really bad meltdowns where I just want to be back to normal [...] I do half days at school [...] go in at like 11am, and I come home and I just, I'm crying [and] 'I just want to be normal again'"* (Mae, 11, LC 8 months)

IE 3. *"[S]he will always try…so she will get up in the morning, she will get herself ready [...] I'm like, 'You are not well,' and she's like, 'But I will try, Mummy.' [...] So she's very resilient, she doesn't ever let anything kind of hold her back [...] if she thinks she can try, she will"* (Evelynn, parent of 8-year-old, LC 13 months)

IE 4. *"[The hardest part is] not being able to go to school or like see people my age, socialise and everything. It's all like online for me now over like social media or messages [...] seeing other people [...] my age that are going out in school or doing all their exams [and] doing lots of things throughout the summer that I would like to be able to do, but I just can't. I think is that's quite hard."* (Gemma, 16, LC 19 months)

For peer review only

For peer review only

Box 2 "School is still like a lot of energy": experiences of returning to school with LC - illustrative interview extracts

IE 1. *"[My daughter] would struggle, do a day [at school] and then she'd be in bed for two days. She'd crash, she couldn't get out of bed. She had pins and needles in her leg every time she stood up, she felt dizzy, nausea […] so [it was like being] on a roller coaster"* (Olivia, parent of 11-year-old, LC 6 months)

IE 2. *"I just feel like ever since I've got [Covid], I've just lost a lot of my drive [for school] just because I'm always in such pain […] constantly taking breaks and being in pain and you can't really concentrate if you were just, like, burning inside"* (Holly, 14, LC 6 months)

IE 3. *"The last three days, my fingers have been really sore and it hurts to bend them and move them. So, [classroom assistant] writes for me"* (Josie, 10, LC 3 months)

IE 4. *"Something's just...blocking my thoughts […] I can't take in English, I can't analyse anything, it's like your sort of chain of thoughts is just completely, you know, broken, like I can't have more than one thought that leads to another one, it's just like my brain is just a cement block and it's just all messed up […] it's so bad not being able to fully focus in class and struggling with the work and then not having the energy to do the homework […]I've never really struggled in school before, I've always enjoyed learning, but […] it really stopped all that [and] just feeling really like stupid […] I couldn't do the work even if it was work that I could do before, [so] then in that way it impacts […] your self-esteem and things like that"* (Erin, 15, LC 6 months)

IE 5. *"[For my son] online learning at home wasn't an option either because he has quite a lot of brain fog so has a lot of difficulty with concentration and was finding learning new things seems to be really difficult, short-term memory, all sorts of things like that were really hard"* (Marissa, parent of 16-year-old, LC 12 months)

IE 6. *"I have a timeout pass [to excuse myself from class] and sometimes I have to go to the toilet [because] I get stressed because everyone else [in class] is working [and] you're like, 'What? I don't understand. What is happening?' and I feel like I get on people's nerves [when] I'm like, 'What?' and [they say] 'Well, you, you probably missed that,'"* (Mae, 11, LC 8 months)

IE 7. *"[My friends] haven't really reacted, mainly they just like ask why I wasn't in school, and formed other friendships"* (Shay, 12, LC 19 months)

IE 8. *"Some subjects I'm behind, but I'm still doing maths, English, science, computer science, I'm still making sure I'm on top of them really […] But I've just left [other] subjects [because] it will take more brain power [and] be un-needed stress"* (Fred, 14, LC 15 months)

Box 3 "It's a mixed bag": Schools' responses to CYP with LC - illustrative interview extracts

IE 1. *"…applying a lot of pressure for us to send her back in and we were saying 'Yeah, we want her to be in school but there's a huge outbreak [of Covid] and... and you're offering her no protection.' The attitude of the headteacher was, 'I'm not going to talk to you, I've answered your questions, I'm following government guidance, I'm not going to talk to you anymore about this,' so she completely closed down the conversation around health and safety*" (Laura, parent of 6-year-old, LC 10 months)

IE 2. *"[All the parents on Long Covid support groups] have the same problem. If the doctor doesn't provide any letters […] it's not that the school doesn't want to support a child, but they need to have some kind of evidence [and] if the paediatrician doesn't recognise that you know, yes the child has a reason for a... tired for a reason they would say, 'Well, I don't see anything why there are... you know all the blood test results come back fine, there is no other reason,' then the school has no backup to say 'Yes, OK we can authorise this.' […] [Once] I'd managed to get a letter from a paediatrician then [the school] said, 'Yes, you know, how many hours can he do? What can he do?'*" (Freja, parent of 12-year-old, LC 8 months, interviewed in May 2022)

IE 3. "*I've got [an Artificial Intelligence-based] robot now which can go into school [when I'm off] […] I do like a day on like a Tuesday and a Thursday on the robot. So, if I'm tired, I can be in my bed, resting and just listening to [what is being taught in class through the robot]. So, I'm still learning stuff*" (Faye, 14, LC 17 months)

IE 4. *"… [because my daughter] was on the first floor [the] school had to move around six classes to be able to get [her wheelchair] into a ground floor classroom […] the school couldn't have done more*" (Jan, parent of 10-year old, LC 3 months)

IE 5. *"[My teachers are] trying to get me back into school […] full-time again, but like it's hard for me […] some of the teachers aren't [supportive] because they're telling my mum they're going to fine her […] if I don't go back full-time […] she'd got a letter from the GP saying that there was something wrong with me and I've been sick [but] I don't think they understand the effect [Long Covid] has on you […] I think they think that I'm not going to school because I don't want to [but] I liked going to school, like I never had a problem with it.*" (Hala, 15, LC 11 months, interviewed in June 2022)

Box 4 Recommendations for schools and healthcare professionals supporting CYP with Long Covid

1. Demonstrate belief, understanding and a willingness to help CYP with LC attempting to return to school and validate their strong desire to return to school and a 'normal life'

2. Recognise the difficulties parents of CYP with LC may face in facilitating school contact with healthcare professionals in the face of pressures on healthcare services, and in future-planning for attendance due to the unpredictable nature of LC

3. Raise awareness and knowledge of LC among the school community (staff and CYP)

4. Communicate regularly with CYP with LC about how they are coping with school alongside their illness

5. Offer a range of adaptations which can be tailored to the individual and changing needs of CYP with LC. For example:
a. Reduced timetables and/or prioritisation of fewer subjects
b. Rest/'time-out' passes
c. Allow the use of lifts and other means of alleviating the physical impacts of moving around school buildings
d. Consider the use of appropriate technology (e.g. Artificial Intelligence-based robots) to facilitate engagement with school

6. Ensure school staff are advised of CYP who have LC and are aware of the adaptations available to them

7. Consult school staff on their experiences of supporting CYP with LC and what they need to facilitate positive engagement with this group of pupils

only

Standards for Reporting Qualitative Research (SRQR)*

http://www.equator-network.org/reporting-guidelines/srqr/

Title and abstract

	Page/line no(s).
**Title** - Concise description of the nature and topic of the study Identifying the study as qualitative or indicating the approach (e.g., ethnography, grounded theory) or data collection methods (e.g., interview, focus group) is recommended	1
**Abstract** - Summary of key elements of the study using the abstract format of the intended publication; typically includes background, purpose, methods, results, and conclusions	2

Introduction

**Problem formulation** - Description and significance of the problem/phenomenon studied; review of relevant theory and empirical work; problem statement	3
**Purpose or research question** - Purpose of the study and specific objectives or questions	3

Methods

**Qualitative approach and research paradigm** - Qualitative approach (e.g., ethnography, grounded theory, case study, phenomenology, narrative research) and guiding theory if appropriate; identifying the research paradigm (e.g., postpositivist, constructivist/ interpretivist) is also recommended; rationale**	3-4
**Researcher characteristics and reflexivity** - Researchers' characteristics that may influence the research, including personal attributes, qualifications/experience, relationship with participants, assumptions, and/or presuppositions; potential or actual interaction between researchers' characteristics and the research questions, approach, methods, results, and/or transferability	4
**Context** - Setting/site and salient contextual factors; rationale**	4
**Sampling strategy** - How and why research participants, documents, or events were selected; criteria for deciding when no further sampling was necessary (e.g., sampling saturation); rationale**	4
**Ethical issues pertaining to human subjects** - Documentation of approval by an appropriate ethics review board and participant consent, or explanation for lack thereof; other confidentiality and data security issues	4
**Data collection methods** - Types of data collected; details of data collection procedures including (as appropriate) start and stop dates of data collection and analysis, iterative process, triangulation of sources/methods, and modification of procedures in response to evolving study findings; rationale**	4

For peer review only - http://bmjopen.bmj.com/site/about/guidelines.xhtml

**Data collection instruments and technologies** - Description of instruments (e.g., interview guides, questionnaires) and devices (e.g., audio recorders) used for data collection; if/how the instrument(s) changed over the course of the study	4
**Units of study** - Number and relevant characteristics of participants, documents, or events included in the study; level of participation (could be reported in results)	4-6
**Data processing** - Methods for processing data prior to and during analysis, including transcription, data entry, data management and security, verification of data integrity, data coding, and anonymization/de-identification of excerpts	4
**Data analysis** - Process by which inferences, themes, etc., were identified and developed, including the researchers involved in data analysis; usually references a specific paradigm or approach; rationale**	4
**Techniques to enhance trustworthiness** - Techniques to enhance trustworthiness and credibility of data analysis (e.g., member checking, audit trail, triangulation); rationale**	4

Results/findings

**Synthesis and interpretation** - Main findings (e.g., interpretations, inferences, and themes); might include development of a theory or model, or integration with prior research or theory	6-8
**Links to empirical data** - Evidence (e.g., quotes, field notes, text excerpts, photographs) to substantiate analytic findings	6-8

Discussion

**Integration with prior work, implications, transferability, and contribution(s) to the field -** Short summary of main findings; explanation of how findings and conclusions connect to, support, elaborate on, or challenge conclusions of earlier scholarship; discussion of scope of application/generalizability; identification of unique contribution(s) to scholarship in a discipline or field	8-10
**Limitations** - Trustworthiness and limitations of findings	

Other

**Conflicts of interest** - Potential sources of influence or perceived influence on study conduct and conclusions; how these were managed	10
**Funding** - Sources of funding and other support; role of funders in data collection, interpretation, and reporting	10

*The authors created the SRQR by searching the literature to identify guidelines, reporting standards, and critical appraisal criteria for qualitative research; reviewing the reference lists of retrieved sources; and contacting experts to gain feedback. The SRQR aims to improve the transparency of all aspects of qualitative research by providing clear standards for reporting qualitative research.

For peer review only

**The rationale should briefly discuss the justification for choosing that theory, approach, method, or technique rather than other options available, the assumptions and limitations implicit in those choices, and how those choices influence study conclusions and transferability. As appropriate, the rationale for several items might be discussed together.

<u>Reference:</u>

O'Brien BC, Harris IB, Beckman TJ, Reed DA, Cook DA. **Standards for reporting qualitative research: a synthesis of recommendations.** *Academic Medicine*, Vol. 89, No. 9 / Sept 2014 DOI: 10.1097/ACM.0000000000000388

For peer review only

For peer review only - http://bmjopen.bmj.com/site/about/guidelines.xhtml

BMJ Open

The impact of Long Covid on the school experiences of children and young people: a qualitative study.

Journal:	*BMJ Open*
Manuscript ID	bmjopen-2023-075756.R1
Article Type:	Original research
Date Submitted by the Author:	28-Jul-2023
Complete List of Authors:	MacLean, Alice; University of Stirling, Institute for Social Marketing and Health<br>Wild, Cervantee; University of Oxford, Nuffield Department of Primary Care Health Sciences<br>Hunt, Kate; University of Stirling, Institute for Social Marketing<br>Nettleton, Sarah; University of York, Sociology<br>Skea, Zoë; University of Aberdeen, Health Services Research Unit<br>Ziebland, Sue; University of Oxford, NDPCHS
**Primary Subject Heading**:	Public health
Secondary Subject Heading:	Qualitative research
Keywords:	COVID-19, Schools, QUALITATIVE RESEARCH, Adolescent

SCHOLARONE™
Manuscripts

I, the Submitting Author has the right to grant and does grant on behalf of all authors of the Work (as defined in the below author licence), an exclusive licence and/or a non-exclusive licence for contributions from authors who are: i) UK Crown employees; ii) where BMJ has agreed a CC-BY licence shall apply, and/or iii) in accordance with the terms applicable for US Federal Government officers or employees acting as part of their official duties; on a worldwide, perpetual, irrevocable, royalty-free basis to BMJ Publishing Group Ltd ("BMJ") its licensees and where the relevant Journal is co-owned by BMJ to the co-owners of the Journal, to publish the Work in this journal and any other BMJ products and to exploit all rights, as set out in our licence.

The Submitting Author accepts and understands that any supply made under these terms is made by BMJ to the Submitting Author unless you are acting as an employee on behalf of your employer or a postgraduate student of an affiliated institution which is paying any applicable article publishing charge ("APC") for Open Access articles. Where the Submitting Author wishes to make the Work available on an Open Access basis (and intends to pay the relevant APC), the terms of reuse of such Open Access shall be governed by a Creative Commons licence – details of these licences and which Creative Commons licence will apply to this Work are set out in our licence referred to above.

Other than as permitted in any relevant BMJ Author's Self Archiving Policies, I confirm this Work has not been accepted for publication elsewhere, is not being considered for publication elsewhere and does not duplicate material already published. I confirm all authors consent to publication of this Work and authorise the granting of this licence.

For peer review only

Title:
The impact of Long Covid on the school experiences of children and young people: a qualitative study.

Author names:

Alice MacLean[1], Cervantee Wild[2], Kate Hunt[1], Sarah Nettleton[3], Zoe Skea[4] and Sue Ziebland[2]

Full institutional mailing addresses:

[1]Institute for Social Marketing and Health, University of Stirling, Stirling, Scotland, FK9 4LA, UK
[2]Health Experiences Research Group, Nuffield Department of Primary Care Health Sciences, University of Oxford, OX2 6GG, UK
[3]Department of Sociology, University of York, Heslington East Campus, York, England, YO10 5GD, UK
[4] Health Service Research Unit, University of Aberdeen, Aberdeen, Scotland, AB25 2DZ, UK

Email addresses:

alice.maclean@stir.ac.uk
cervantee.wild@phc.ox.ac.uk
kate.hunt@stir.ac.uk
sarah.nettleton@york.ac.uk
z.skea@abdn.ac.uk
sue.ziebland@phc.ox.ac.uk

For peer review only

Abstract

Objectives To explore the impact of Long Covid on the school experiences of children and young people (CYP).

Design Qualitative study using narrative interviews.

Participants 22 CYP (aged 10-18 years, 15 female) with Long Covid and 15 parents/caregivers (13 female) of CYP (aged 5-18 years) with Long Covid.

Setting Interviews were conducted between October 2021 and July 2022 via online video call or telephone. Recruitment routes included social media, LC support groups, clinicians, community groups and snowballing.

Results Three key findings were identified. Finding 1: Going to school is a valued part of CYP's lives and participants viewed educational attainment as important for their future trajectories. Returning to school full-time was highlighted as a key part of regaining 'normal life'. Finding 2: Attending school (in-person or online) with Long Covid is extremely difficult; even a gradual return required CYP to balance the impact of being at and engaging with school, with the need to manage symptoms to prevent relapse. Often this meant prioritising school and rest over other aspects of their lives. Finding 3: School responses to CYP with Long Covid were reported to be mixed and hampered by difficulties communicating with healthcare professionals during the pandemic and a lack of awareness of Long Covid among healthcare and education professionals. Participants viewed supportive school responses as staff believing, understanding and taking them seriously, alongside schools offering tailored and flexible adaptations which allowed engagement with school while limiting any deterioration of symptoms.

Conclusions This study describes how Long Covid affects the school experiences of CYP and generates recommendations for supportive school responses alongside supportive healthcare professionals. Further research could explore the approaches that facilitate a successful return to school for CYP with Long Covid and investigate education professionals' perspectives on support they require to positively engage with returning pupils.

Strengths and limitations of this study

- To our knowledge, this is the first qualitative study to explore the impact of LC on CYP's experiences of school.
- This study utilises rigorous methods and draws on rich accounts from a diverse sample of CYP with LC, and parents of CYP with Long Covid, across the UK.
- A limitation of the study is that it does not include the perspectives of education professionals on their experiences of engaging with CYP with LC and the support they need to do this.
- The composition of our sample prevented a systematic comparison of accounts by age, gender, ethnicity or social class, thus preventing investigation of whether CYP with LC experience similar expectations around responding to illness with stoicism, control and independence (especially while at school) which previous research has highlighted.

For peer review only

INTRODUCTION

Children and young people (CYP) infected with SARS-CoV-2 are at lower risk of hospitalisation and mortality compared to adults (1) but, like adults, can experience symptoms which persist long after initial infection (2-4). These ongoing symptoms are variously described as post-COVID-19 syndrome, post-COVID-19 condition and post-acute sequelae of SARS-CoV-2 (PASC) or Long Covid (LC), the name first used by adults with persisting symptoms (5). There is an increasing research focus on LC in adults (e.g. 6-9), but limited high-quality evidence on CYP and ongoing uncertainties, including the prevalence, risk factors, mechanisms and effective management of LC in CYP (10-13). Here, we describe accounts from CYP and parents of the impacts of LC on education and school-related challenges and recommend ways that schools could support CYP with LC.

Long Covid has been described as a "frequent legacy of acute SARS-CoV-2 infection, affecting over 10% of patients" (7 p.1891) with wide-ranging symptoms. Its clinical case definition in adults describes: "a history of probable or confirmed SARS-CoV-2 infection, usually 3 months from the onset of Covid-19 with symptoms…that last for at least 2 months and cannot be explained by an alternative diagnosis" (14) and research suggests CYP have comparable symptom experiences to adults (15). Children and adults report: many persistent symptoms affecting multiple body systems (11), with fatigue, headache, cognitive impairment and shortness of breath being common (2, 16-18); and symptoms that fluctuate in severity (11), with new symptoms emerging potentially months after initial infection. A higher prevalence of persistent symptoms has been reported in females (13, 19). Accurately measuring symptoms of Long Covid over time is challenging given the emergence of new variants of Covid-19, the potential impact of vaccines and the difficulty of ruling out the influence of other viral infections (3, 20).

Research into the social and academic impacts of LC on CYP is lacking and existing evidence is mixed. A Spanish study followed (for five months) 50 CYP with LC (defined as ongoing symptoms for 12+ weeks) and reported that 18% were unable to attend school, 34% had a reduced school schedule, 66% showed decreased school performance and 68% had stopped extracurricular activities (15). Systematic reviews have highlighted heterogeneous findings relating to the impact of LC on CYP's lives. Pellegrino et al (12) included five studies which reported limitation in daily function affecting school attendance (in 10.5 to 58.9% of participants across the studies). Franco et al (21) reviewed 25 studies which included wellbeing and recovery outcomes in CYP, five of which reported outcomes relating to 'changes in work/occupation and study (school attendance)'. The authors suggested "most children with persistent symptoms reported no substantial impairment in their school functioning at 3–6-month follow-ups" (p.12). However, it has been noted that the evidence in CYP is limited, heterogeneous and largely based on low-quality studies (12).

We found no qualitative studies on the impacts of LC on CYP's school experiences. However, research with CYP with chronic fatigue syndrome (CFS)/myalgic encephalomyelitis (ME), a condition with some similarities to LC (22, 23), highlights detrimental impacts on social, emotional and academic aspects of schooling. For CYP with CFS/ME, disrupted schooling "has a significant impact on the self [resulting] in a shift from a perceived normal trajectory of academic achievement and independence to one that is uncertain" (24 p.10), and some educational support and adaptations underpinned by evidence-based awareness-raising in schools have been recommended (25-28).

METHODS

This paper draws on narrative interviews undertaken to improve understanding of the experience of LC in households with CYP from diverse backgrounds. In this analysis we draw on interviews with CYP (aged

10-18 years) with LC and parents/caregivers of CYP (aged 5-18 years) with LC, some of whom had LC themselves.

Patient and public involvement

An advisory panel, including patient and public involvement representatives with lived experience of LC or of caring for a child or young person with LC, had input into all aspects of the study conduct, including content of the interview topic guide and recruitment methods.

Recruitment and sampling

Recruitment routes included social media, LC support groups, clinicians, community groups and snowballing. We aimed for maximum variation sampling (29), to capture diversity by age, gender, ethnicity, geographical location, and social class. Potential participants were eligible if they, or the person they cared for, had self-identified ongoing symptoms 12+ weeks after initial Covid-19 infection. CYP were eligible if they were 10 years or over. Parents were eligible if their child with LC was five years or over. Age-appropriate information sheets were provided to potential participants.

Data collection

Narrative interviews (n=37) were conducted (between October 2021 and July 2022) via online video call or telephone, and were video and/or audio recorded, depending on participant preference. Verbal consent was recorded at the start of their interview. Those under 16 years gave assent and their parent/caregiver gave proxy consent. Participants were offered a £30 voucher to thank them for sharing their time and experiences.

Interviews began with an open narrative where participants were asked to recount events since they/their child first experienced signs of Covid. The second part used topic guides (different for CYP and parent interviews) with prompts, including questions about how Long Covid had affected school (see supplementary file). Interviews typically lasted between 25 and 90 minutes, with some conducted over multiple shorter sessions to accommodate participant fatigue or other symptoms.

Data analysis

Interviews were transcribed verbatim, checked for accuracy and imported into NVivo (March 2020 version) to aid organisation and coding of data. We used thematic analysis (30) to inductively code the data. After initial familiarisation with transcripts, we developed a coding framework of broad themes, which was refined throughout the coding process. Three researchers coded the transcripts (CW, ZS, SN). All data coded to the broad 'school/education' theme were then further analysed (by AM) using the mind-mapping 'one sheet of paper' (OSOP) technique (31). This process generated three sub-themes, as described below with interview extracts (IE) to illustrate the range of views (longer extracts are presented in boxes 1-3). All names are pseudonyms. When quoting a parent who spoke in their child's interview, we use the convention 'mother of Gemma (16 years old, LC 19-24 months)'.

Ethics

Ethical approval was granted by Berkshire Ethics Committee (12/SC/0495).

RESULTS

Participants

Table 1 displays the characteristics of the participants included in this analysis. Of the 15 parents of a child/young person with LC interviewed, five also had LC themselves.

For peer review only

Table 1 Participant characteristics (n=37)			
**Age (years)**		**Sex**	
10-12	8	Male	9
13-18	14	Female	28
19-30	0		
31-40	6		
41-50	7		
51+	1		
Missing	1		
**Ethnicity**		**Time child affected by LC symptoms (months)**	
White British	21	0-6	12
White Other	3	7-12	15
British Asian	8	13-18	4
Black Other	1	19-24	5
Mixed race	1	25+	1
Other	3		

In interviews with CYP, some parents chose to be present, either sitting beside their child or elsewhere in the room while the interview was conducted. When parents were present, some spoke very little in the interview and others contributed significantly. The sample included four families where more than one member took part individually, and one family contributed three separate interviews (two with children with LC and one with their parent).

Analysis identified three key themes: CYP's desire to get back to school; their experiences of being in school whilst still affected by Long Covid; and schools' responses to their illness.

"*I just want to be normal again*" (Mae, 11 years old, LC 7-12 months): education as a normal and valued aspect of life for CYP

Most CYP's accounts indicated that they had been too ill to attend school regularly or undertake online learning and some were not attending school at all when interviewed. GP and hospital appointments also caused frequent school absences. Analysis revealed a strong desire for their lives to return to the way they were before having Covid and CYP spoke about being able to go back to school as a major means of regaining some normality (see Box 1-IE 1&2). Children's eagerness to return to school was also evident in parents' accounts (see Box 1-IE 3).

Box 1 "*I just want to be normal again*": education as a normal and valued aspect of life for CYP - illustrative interview extracts

IE 1. "*I just want to go back to school. Right, I know a lot of kids can't say that, but [I want to] just go back to my normal routine*" (Holly, 14 years old, LC 0-6 months)

IE 2. "*I have really bad meltdowns where I just want to be back to normal […] I do half days at school […] go in at like 11am, and I come home and I just, I'm crying [and] 'I just want to be normal again'*" (Mae, 11 years old, LC 7-12 months)

IE 3. *"[S]he will always try…so she will get up in the morning, she will get herself ready […] I'm like, 'You are not well,' and she's like, 'But I will try, Mummy.' […] So she's very resilient, she doesn't ever let anything kind of hold her back […] if she thinks she can try, she will"* (Evelynn, parent of 8-year-old, LC 13-18 months)

IE 4. *"[The hardest part is] not being able to go to school or like see people my age, socialise and everything. It's all like online for me now over like social media or messages […] seeing other people […] my age that are going out in school or doing all their exams [and] doing lots of things throughout the summer that I would like to be able to do, but I just can't. I think is that's quite hard."* (Gemma, 16 years old, LC 19-24 months)

Expressing their desire to return to school contradicts common stereotyping of children, and particularly teenagers, as lazy or reluctant to engage with school. Faye (14 years old, LC 13-18 months) said "*I would give so much to go back to school full-time. I miss it a lot.*" CYP's accounts portrayed school absence as making them stand out from their peers, going against the 'normality' of full-time school for people their age. Frequent or extended absences were described as stressful and isolating, leading to feelings of being left behind academically and socially (see Box 1-IE 4). The unpredictable and variable nature of symptoms was particularly distressing because CYP did not know how long disruption to their schooling would last. Fred (14 years old, LC 13-18 months) said *"It's never ending […] you're in a maze and you turn around the corner and 'Oh, this is going to be the end,' but it's a dead end, that's what it feels like."*

"*School is still a lot of energy*" (Alana, 13 years old, LC 7-12 months): experiences of returning to school with LC

For many CYP we talked to, going back to school had not represented the hoped for 'return to normal' and they commonly highlighted extreme fatigue.

> "*after school, I would come home [and] sleep for ages [and] I've never done that before*" (Faye, 14 years old, LC 13-18 months).
>
> "*I couldn't really do anything [with friends] at break. I was just resting. I struggled going up the stairs. I can't do PE. Yeah, I just feel tired after every lesson*" (Rory, 13 years old, LC 7-12 months).

Attempts to return to school invariably led to "*crashes*", "*huge relapses*" or feeling "*exhausted*", followed by needing more time off to recover (see Box 2-IE 1).

For peer review only - http://bmjopen.bmj.com/site/about/guidelines.xhtml

Box 2 "*School is still a lot of energy*": experiences of returning to school with LC - illustrative interview extracts

IE 1. "*[My daughter] would struggle, do a day [at school] and then she'd be in bed for two days. She'd crash, she couldn't get out of bed. She had pins and needles in her leg every time she stood up, she felt dizzy, nausea […] so [it was like being] on a roller coaster*" (Olivia, parent of 11-year-old, LC 0-6 months)

IE 2. "*I just feel like ever since I've got [Covid], I've just lost a lot of my drive [for school] just because I'm always in such pain […] constantly taking breaks and being in pain and you can't really concentrate if you were just, like, burning inside*" (Holly, 14 years old, LC 0-6 months)

IE 3. "*The last three days, my fingers have been really sore and it hurts to bend them and move them. So, [classroom assistant] writes for me*" (Josie, 10 years old, LC 0-6 months)

IE 4. "*Something's just...blocking my thoughts […] I can't take in English, I can't analyse anything, it's like your sort of chain of thoughts is just completely, you know, broken, like I can't have more than one thought that leads to another one, it's just like my brain is just a cement block and it's just all messed up […] it's so bad not being able to fully focus in class and struggling with the work and then not having the energy to do the homework […]I've never really struggled in school before, I've always enjoyed learning, but […] it really stopped all that [and] just feeling really like stupid […] I couldn't do the work even if it was work that I could do before, [so] then in that way it impacts […] your self-esteem and things like that*" (Erin, 15 years old, LC 0-6 months)

IE 5. "*[For my son] online learning at home wasn't an option either because he has quite a lot of brain fog so has a lot of difficulty with concentration and was finding learning new things seems to be really difficult, short-term memory, all sorts of things like that were really hard*" (Marissa, parent of 16-year-old, LC 7-12 months)

IE 6. "*I have a timeout pass [to excuse myself from class] and sometimes I have to go to the toilet [because] I get stressed because everyone else [in class] is working [and] you're like, 'What? I don't understand. What is happening?' and I feel like I get on people's nerves [when] I'm like, 'What?' and [they say] 'Well, you, you probably missed that,'*" (Mae, 11 years old, LC 7-12 months)

IE 7. "*[My friends] haven't really reacted, mainly they just like ask why I wasn't in school, and formed other friendships*" (Shay, 12 years old, LC 19-24 months)

IE 8. "*Some subjects I'm behind, but I'm still doing maths, English, science, computer science, I'm still making sure I'm on top of them really […] But I've just left [other] subjects [because] it will take more brain power [and] be un-needed stress*" (Fred, 14 years old, LC 13-18 months)

Navigating school buildings was difficult and participants said it made symptoms worse.

> "*school is still a lot of energy because I go to a very big school […] so that's a lot of walking about*" (Alana, 13 years old, LC 7-12 months).

> "*I've got third floor, second floor, first floor, the ground floor, I have to go up and down stuff [and] I get tired*" (Rohaan, 12 years old, LC 0-6 months).

> "*[My classroom] used to be upstairs and that was the hardest bit because before I stopped walking [and got a wheelchair], I had to go upstairs and my knees were so sore*" (Josie, 10 years old, LC 0-6 months)).

As well as fatigue, other physical symptoms impacted participants' ability to learn (see Box 2-IE 2&3).

Many highlighted difficulties caused by cognitive impairment, such as Shay (12 years old, LC 19-24 months) who said "*I can't concentrate with reading*" and Fred (14 years old, LC 13-18 months) who stated "*I sometimes get brain fog, I'm sometimes just lost*". Those who previously enjoyed school described these difficulties as particularly distressing (see Box 2-IE 4). For many, learning online from home was not an appropriate solution. Gemma (16 years old, LC 19-24 months) who was no longer going to school when interviewed, said "*[for two months] I was trying to do a full week at school from home while not feeling well and I just couldn't cope with it, so I was removed from all my classes*". Those with brain fog and fatigue struggled to follow online learning (see Box 2-IE 5). There were also social and emotional difficulties involved in returning to school. Some found it distressing and isolating to feel they were falling behind their peers academically, and some described how their absences from school contributed to the disintegration of friendships (see Box 2-IE 6&7).

CYP who were able to attend school, at least part-time, struggled to make a successful return while managing their symptoms. Secondary school pupils spoke about being aware of the importance of education for their future and wanting to do well in exams to keep further education and career options open ("*I want to get good GCSEs*" (Layla, 14 years old, LC 7-13 months)). Balancing the impact of working hard at school with managing their symptoms and preventing relapses was difficult. Sacrifices and prioritisation were often recounted, such as concentrating on fewer school subjects (see Box 2-IE 8) or restricting social activities to conserve energy for going to school. Erin (15 years old, LC 0-6 months) said "*I'm not doing anything outside of school really […] I go to school, I go to sleep, […] which is difficult*".

"*It's a mixed bag*" (Fred, 14 years old, LC 13-18 months): Schools' responses to CYP with LC

Parents spoke about alerting their child's school to the health challenges they were experiencing and explaining their absences. This could be difficult due to the varied and unpredictable nature of symptoms, as indicated by Freja (parent of 12-year-old, LC 7-12 months): "*I said to [son's school], 'No, he doesn't have fever, he is just exhausted, I can't send him to school, I'm sorry,' so of course [the school is] concerned*". One parent, whose child caught Covid in Winter 2021, said she had tried to explain LC to her daughter's school "*because it's new to them as well*" (Angela, parent of 8-year-old, LC for 0-6 months). Parents understood schools require explanations for absences, ideally "backed-up" by validation from healthcare professionals. However, the pandemic had made it harder to facilitate communication between healthcare professionals and schools.

When Covid cases were high in school, some parents preferred their child to stay away to avoid reinfection:

> "*cases are so high [that] I've got no interest in sending [my children to school]*" (Izzy, parent of 12-year-old, LC 19-24 months).

For peer review only

> *"[son] went back [to school] again [and] then we had to miss the last couple of days [before Christmas holidays] because cases were going through the roof*" (Ross, parent of 13-year-old, LC 19-24 months)).

A paediatrician's reported advice that school was "*absolutely the best place*" for her daughter, was unwelcome to a parent who felt her concerns about reinfection and its impacts were dismissed:

> "*I kept saying, 'But what happens if she goes back to school and gets Covid [again] on top of how she's feeling?'*" (mother of Gemma, 16 years old, LC 19-24 months).

Another mother, whose daughter caught Covid in Spring 2021, said school had ignored her fears about the risk of reinfection and she felt they were applying pressure to send her daughter back to school (see Box 3-IE 1).

For peer review only

Box 3 "*It's a mixed bag*": Schools' responses to CYP with LC - illustrative interview extracts

IE 1. *"…applying a lot of pressure for us to send her back in and we were saying 'Yeah, we want her to be in school but there's a huge outbreak [of Covid] and... and you're offering her no protection.' The attitude of the headteacher was, 'I'm not going to talk to you, I've answered your questions, I'm following government guidance, I'm not going to talk to you anymore about this,' so she completely closed down the conversation around health and safety*" (Laura, parent of 6-year-old, LC 7-12 months)

IE 2. *"[All the parents on Long Covid support groups] have the same problem. If the doctor doesn't provide any letters […] it's not that the school doesn't want to support a child, but they need to have some kind of evidence [and] if the paediatrician doesn't recognise that you know, yes the child has a reason for a... tired for a reason they would say, 'Well, I don't see anything why there are... you know all the blood test results come back fine, there is no other reason,' then the school has no backup to say 'Yes, OK we can authorise this.' […] [Once] I'd managed to get a letter from a paediatrician then [the school] said, 'Yes, you know, how many hours can he do? What can he do?'*" (Freja, parent of 12-year-old, LC 7-12 months, interviewed in Spring 2022)

IE 3. "*I've got [an Artificial Intelligence-based] robot now which can go into school [when I'm off] […] I do like a day on like a Tuesday and a Thursday on the robot. So, if I'm tired, I can be in my bed, resting and just listening to [what is being taught in class through the robot]. So, I'm still learning stuff*" (Faye, 14 years old, LC 13-18 months)

IE 4. *"… [because my daughter] was on the first floor [the] school had to move around six classes to be able to get [her wheelchair] into a ground floor classroom […] the school couldn't have done more*" (Jan, parent of 10-year old, LC 0-6 months)

IE 5. *"[My teachers are] trying to get me back into school […] full-time again, but like it's hard for me […] some of the teachers aren't [supportive] because they're telling my mum they're going to fine her […] if I don't go back full-time […] she'd got a letter from the GP saying that there was something wrong with me and I've been sick [but] I don't think they understand the effect [Long Covid] has on you […] I think they think that I'm not going to school because I don't want to [but] I liked going to school, like I never had a problem with it.*" (Hala, 15 years old, LC 7-12 months, interviewed in Summer 2022)

A perceived lack of integration of care across health and education settings was compounded by the fact that LC in CYP was a new condition that was neither widely recognised nor well understood. Parents suggested that the absence of a formal diagnosis meant schools and education professionals were limited in the support and adaptations they could offer (see Box 3- IE2). Even when contact was made between health and education practitioners, it was then hard for parents and CYP to plan how much and how often they could attend school because symptoms were unpredictable, varying day-to-day or week-to-week and activity needed to be balanced against potential relapses.

Other participants recounted positive experiences once a link had been made between school and a healthcare professional who suggested ways to manage symptoms at school (e.g., attending part-time, not doing physical education, frequent rest breaks). Some schools were described as responsive, assertive,

supportive, and flexible, by putting various adjustments in place (see Box 3-IE 3&4). Parents and CYP also mentioned understanding and supportive teachers:

> "*My art teacher has been really good [and] worked out what work absolutely needed to be done [and] came up with the idea of basing my whole art project around Long Covid*" (Molly, 16 years old, LC 0-6 months).

> *"[The teacher's] wife had chronic fatigue syndrome so he's really understanding and he's amazing with [my daughter]"* (Olivia, parent of 11-year old, LC 0-6 months).

CYP and parents wanted to feel that school staff believed them and took the impact of LC seriously. This was the case for Evelynn (parent of 8-year-old child, LC 13-18 months) who said *"[her teachers] are really flexible if she needed a break, she can go and sit down: there's no questions asked*".

However, a few parents and CYP said that schools had not implemented systems to facilitate reintegration.

> "*I had to tell [my teachers about Long Covid] myself [because] all school told them was that I was going part-time*" (Faye, 14 years old, LC 17-24 months).

> *"[During PE] they forgot I couldn't do stuff [and] I just had to sort of watch them do fun stuff*" (Rory, 13 years old, LC 7-13 months).

Others, including some interviewed in 2022, described a lack of awareness and understanding of LC among school staff. Fred (14 years old, LC 13-18 months), interviewed in Spring 2022, described his teachers' responses as a "*mixed bag*"; some understanding teachers said, "*don't push yourse*lf" while others were "*the opposite and want me to do the same things and the same tests [as other pupils]*". Even when healthcare professionals had written to the school, there were still accounts of disbelief, a lack of understanding and even threats of fines for non-attendance (see Box 3-IE 5).

DISCUSSION

Our findings highlight the importance of school to CYP with LC and how returning to school was central to CYP's much hoped for 'return to normal'. CYP described absences from school as stressful and isolating and placed high importance on returning to school full-time. However, re-engaging with school could lead to relapses, and further absences, meaning CYP had to learn how to deal with the demands of school without pushing themselves to "crashing" point. Often this meant prioritising school and rest over all other aspects of life. The various school responses to LC that participants described have highlighted the importance of validation of CYP's experience of LC by healthcare professionals and informed recommendations for supporting CYP with LC at school to minimise adverse educational, social and mental health sequelae of having LC in childhood and adolescence (see Box 4). These recommendations for practice foreground the lessons arising from dealing with the ongoing symptoms of COVID-19 in children and young people and highlight ways to respond to other long-term health conditions and the potential impacts of future pandemics on school pupils.

For peer review only

Box 4 Recommendations for schools and healthcare professionals supporting CYP with Long Covid

1. Show that you believe, understand and are willing to help CYP with LC attempting to return to school and validate their strong desire to return to school and a 'normal life'

2. Recognise the difficulties parents of CYP with LC may face in facilitating school contact with healthcare professionals in the face of pressures on healthcare services, and in future-planning for attendance due to the unpredictable nature of LC

3. Raise awareness and knowledge of LC among the school community (staff and CYP)

4. Communicate regularly with CYP with LC about how they are coping with school alongside their illness

5. Offer a range of adaptations which can be tailored to the individual and changing needs of CYP with LC. For example:
a. Reduced timetables and/or prioritisation of fewer subjects
b. Rest/'time-out' passes
c. Allow the use of lifts and other means of alleviating the physical impacts of moving around school buildings
d. Consider the use of appropriate technology (e.g. Artificial Intelligence-based robots) to facilitate engagement with school

6. Ensure school staff are advised of CYP who have LC and are aware of the adaptations available to them

7. Consult school staff on their experiences of supporting CYP with LC and what they need to facilitate positive engagement with this group of pupils

To our knowledge, this is the first qualitative study to explore the impact of LC on CYP's experiences of school. A further strength of our study is that it utilises rigorous methods and draws on rich accounts from a diverse sample of CYP with LC across the UK. A limitation is that it does not include the perspectives of education professionals on their experiences of engaging with CYP with LC and the support they need to do this. The composition of our sample prevented a systematic comparison of accounts by age, gender, ethnicity or social class, thus preventing investigation of whether CYP with LC experience similar expectations around responding to illness with stoicism, control and independence (especially while at school) which previous research has highlighted (32). It is also important to acknowledge that participants' accounts may have been impacted by the fact that they were being filmed/audio-recorded for a website. However, all participants were given the option of safeguarding their identity by making their data fully anonymous.

There are few existing studies with which to compare our findings, although there are some parallels with studies of the impact of CFS/ME and other long-term health conditions on school experiences, particularly the importance of school and its centrality to CYP's hopes to return to a 'normal' trajectory (24, 27, 33, 34). School absence because of LC marked CYP out from peers and they reported feeling stressed, isolated (35, 36) and worried about falling behind academically and socially. These negative emotions were

For peer review only - http://bmjopen.bmj.com/site/about/guidelines.xhtml

compounded by not knowing how long this 'break from normality' and uncertain trajectory would continue, as has been reported for CYP with CFS/ME, juvenile idiopathic arthritis and other long-term conditions (24, 35, 36). Echoing the "ripple effect" of CFS/ME on CYP's social, emotional and academic functioning (25), our findings demonstrate the impact of LC on CYP's participation in many aspects of school life, highlighting their distress about their inability to fully reintegrate with peers in the way they had hoped. As CYP placed a high value on education and often prioritised school (and rest) over all other aspects of their lives, there is a need to find ways of helping CYP with LC and other long-term conditions to minimise school demands so that they can also participate in other aspects of their lives (28). As reported for CYP with CFS/ME (27, 28), we found that schools' responses were experienced as particularly supportive when participants felt believed and understood by school staff (often following validation of the CYP's condition by a healthcare professional) and when schools instigated tailored and flexible adaptations to help CYP engage with school while preventing relapse. The recommendations for healthcare and education professionals stemming from our findings are underpinned by values such as empathy, respect, openness and flexibility. They echo the "approaches of positive schools" and key actions in the role of schools and teachers in engaging young people with health conditions outlined by Hopkins et al (37 p.32).

A notable difference from research on other illnesses is that LC emerged as a new condition during a global pandemic. There is an expanding literature on the processes that adults with LC have gone through to recognise their symptoms as legitimate and worthy of investigation, support and treatment (5, 8, 32). However, little is known about equivalent experiences of CYP with LC and their parents/caregivers. Indeed, it has been suggested that resistance to recognising Long Covid as a clinical entity has been even more pronounced for CYP (18). Research on paediatric CFS reports that CYP and parents still "often report feeling misunderstood and disbelieved by medical and educational services" despite CFS being a defined diagnostic entity since 1991 (38 p.12). Studies of CYP with CFS/ME highlight the importance of diagnosis and healthcare professional validation in legitimising the illness (27, 28). Our findings suggest that harnessing this "power of diagnosis" has been especially problematic for CYP with LC due to the novelty of LC and recency of its emergence within a time of unprecedented pressures on healthcare services, which limited parents' ability to facilitate links between healthcare and education professionals. While Simila et al (28) found that online teaching was perceived as helpful by CYP with CFS/ME during periods of lockdown, this was not always the case for CYP with LC in our study. Again, this highlights the need for further research on how educational adaptations can be tailored to the ways that LC symptoms impact upon CYP's abilities to learn to minimise adverse health, social and educational outcomes in later life. For some parents in our study, a further tension existed around whether school was a safe place for CYP with LC, particularly when high case numbers in schools fuelled concerns that reinfection with Covid-19 might exacerbate or prolong LC symptoms. This novel aspect of our findings also requires further exploration. On the whole, it is important to learn from experiences of the COVID-19 pandemic in order to inform responses to future pandemics.

Our findings suggest ways that schools can support CYP with LC and highlight the need to raise awareness of LC among healthcare and education professionals. However, there is a need for further research on appropriate educational and social adaptations for CYP with LC, and increased knowledge and understanding of LC in schools and the most supportive ways that schools can respond. Further research might explore experiences by age, gender, social class and ethnicity to identify where interventions could be focussed. Research with educational professionals could investigate how best to facilitate positive engagement with CYP with LC and their parents. Overall, it is important that CYP with LC are supported to engage with school in ways that facilitate recovery and minimise the impact of LC on their lives, physical and mental health and education longer term.

For peer review only

FUNDING STATEMENT

This work is independent research funded by the National Institute for Health and Care Research (NIHR) (COV-LT2-0005). The views expressed in this publication are those of the author(s) and not necessarily those of NIHR or The Department of Health and Social Care.

COMPETING INTERESTS

None declared.

CONTRIBUTORS

SZ and KH developed the study, with input from CW, SN, ZS, AM and the research advisory group which included CYP with LC and parents of CYP with LC. CW led the day-to-day management of the project, supported by SZ, KH, SN, AM and ZS. The qualitative interviews were conducted by CW, SN, KH, AM and ZS; all are highly experienced qualitative researchers. CW, ZS and SN coded the interview transcripts. AM further analysed all data relating to school/education using the mind-mapping 'one sheet of paper' (OSOP) technique. AM drafted the manuscript, and all authors provided critical comments on drafts, and read and approved the final manuscript. All authors contributed to revisions of the paper.

PATIENT CONSENT FORM

Not applicable.

DATA SHARING STATEMENT

The University of Oxford holds the copyright for the full interview transcripts and may grant data sharing permission upon request.

ACKNOWLEDGEMENTS

We would like to thank all the children, young people and parents who took part in interviews, especially as many of our participants were still very affected by their Long Covid symptoms and had limited physical and cognitive resources. We would like to thank the funders of our research and our colleagues at the Health Experiences Research Group in Oxford. Thank you especially to Dr Anna Dowrick for helpful comments on an earlier draft.

REFERENCES

1. Chua PEY, Shah SU, Gui H, et al. Epidemiological and clinical characteristics of non-severe and severe pediatric and adult COVID-19 patients across different geographical regions in the early phase of pandemic: a systematic review and meta-analysis of observational studies. *J Investig Med* 2021;69(7):1287-96. doi: 10.1136/jim-2021-001858 [published Online First: 20210616]
2. Behnood SA, Shafran R, Bennett SD, et al. Persistent symptoms following SARS-CoV-2 infection amongst children and young people: A meta-analysis of controlled and uncontrolled studies. *J Infect* 2022;84(2):158-70. doi: 10.1016/j.jinf.2021.11.011 [published Online First: 20211120]
3. Kikkenborg Berg S, Palm P. Playing catch with long COVID - understanding a new disease. *Lancet Reg Health Eur* 2023;25 doi: https://doi.org/10.1016/j.lanepe.2022.100568
4. Ludvigsson JF. Case report and systematic review suggest that children may experience similar long-term effects to adults after clinical COVID-19. *Acta Paediatr* 2021;110(3):914-21. doi: 10.1111/apa.15673 [published Online First: 20201203]
5. Callard F, Perego E. How and why patients made Long Covid. *Soc Sci Med* 2021;268:113426. doi: 10.1016/j.socscimed.2020.113426 [published Online First: 20201007]

For peer review only

6. Macpherson K, Cooper K, Harbour J, et al. Experiences of living with long COVID and of accessing healthcare services: a qualitative systematic review. *BMJ Open* 2022;12(1):e050979. doi: 10.1136/bmjopen-2021-050979 [published Online First: 20220111]
7. Mantovani A, Morrone MC, Patrono C, et al. Long Covid: where we stand and challenges ahead. *Cell Death Differ* 2022;29(10):1891-900. doi: 10.1038/s41418-022-01052-6 [published Online First: 20220907]
8. Rushforth A, Ladds E, Wieringa S, et al. Long Covid - The illness narratives. *Soc Sci Med* 2021;286:114326. doi: 10.1016/j.socscimed.2021.114326 [published Online First: 20210819]
9. Subramanian A, Nirantharakumar K, Hughes S, et al. Symptoms and risk factors for long COVID in non-hospitalized adults. *Nat Med* 2022;28(8):1706-14. doi: 10.1038/s41591-022-01909-w [published Online First: 20220725]
10. Franco JVA, Garegnani LI, Oltra GV, et al. Long-Term Health Symptoms and Sequelae Following SARS-CoV-2 Infection: An Evidence Map. *Interntional Journal of Environmental Research and Public Health* 2022(a);19 doi: https://doi.org/10.3390/ijerph19169915
11. Lopez-Leon S, Wegman-Ostrosky T, Ayuzo Del Valle NC, et al. Long-COVID in children and adolescents: a systematic review and meta-analyses. *Sci Rep* 2022;12(1):9950. doi: 10.1038/s41598-022-13495-5 [published Online First: 20220623]
12. Pellegrino R, Chiappini E, Licari A, et al. Prevalence and clinical presentation of long COVID in children: a systematic review. *Eur J Pediatr* 2022;181(12):3995-4009. doi: 10.1007/s00431-022-04600-x [published Online First: 20220915]
13. Stephenson T, Shafran R, Ladhani SN. Long COVID in children and adolescents. *Curr Opin Infect Dis* 2022;35(5):461-67. doi: 10.1097/QCO.0000000000000854 [published Online First: 20220803]
14. WHO. A clinical case definition of post COVID-19 condition by a Delphi consensus 2021 [updated 6 October 2021. Available from: https://www.who.int/publications/i/item/WHO-2019-nCoV-Post_COVID-19_condition-Clinical_case_definition-2021.1 accessed 16th November 2022.
15. Gonzalez-Aumatell A, Bovo MV, Carreras-Abad C, et al. Social, Academic, and Health Status Impact of Long COVID on Children and Young People: An Observational, Descriptive, and Longitudinal Cohort Study. *Children (Basel)* 2022;9(11) doi: 10.3390/children9111677 [published Online First: 20221031]
16. Aiyegbusi OL, Hughes SE, Turner G, et al. Symptoms, complications and management of long COVID: a review. *J R Soc Med* 2021;114(9):428-42. doi: 10.1177/01410768211032850 [published Online First: 20210715]
17. Chen C, Haupert SR, Zimmermann L, et al. Global Prevalence of Post-Coronavirus Disease 2019 (COVID-19) Condition or Long COVID: A Meta-Analysis and Systematic Review. *J Infect Dis* 2022;226(9):1593-607. doi: 10.1093/infdis/jiac136
18. Izquierdo-Pujol J, Moron-Lopez S, Dalmau J, et al. Post COVID-19 Condition in Children and Adolescents: An Emerging Problem. *Front Pediatr* 2022;10:894204. doi: 10.3389/fped.2022.894204 [published Online First: 20220511]
19. Kikkenborg Berg S, Palm P, Nygaard U, et al. Long COVID symptoms in SARS-CoV-2-positive children aged 0-14 years and matched controls in Denmark (LongCOVIDKidsDK): a national, cross-sectional study. *Lancet Child Adolesc Health* 2022;6(9):614-23. doi: 10.1016/S2352-4642(22)00154-7 [published Online First: 20220623]
20. Sakurada Y, Otsuka Y, Tokumasu K, et al. Trends in Long COVID Symptoms in Japanese Teenage Patients. *Medicina* 2023;59 doi: https://www.mdpi.com/1648-9144/59/2/261
21. Franco JVA, Garegnani LI, Oltra GV, et al. Short and Long-Term Wellbeing of Children following SARS-CoV-2 Infection: A Systematic Review. *Int J Environ Res Public Health* 2022(b);19(21) doi: 10.3390/ijerph192114392 [published Online First: 20221103]

For peer review only

22. Siberry VGR, Rowe PC. Pediatric Long Covid and Myalgic Encephalomylitis/Chronic Fatigue Syndrome. Overlaps and Opportunities. *The Pediatric Infectious Diseases Journal* 2022;41(4)
23. Wong TL, Weitzer DJ. Long COVID and Myalgic Encephalomyelitis/Chronic Fatigue Syndrome (ME/CFS)—A Systemic Review and Comparison of Clinical Presentation and Symptomatology. *Medicina* 2021;57
24. Parslow RM, Harris S, Broughton J, et al. Children's experiences of chronic fatigue syndrome/myalgic encephalomyelitis (CFS/ME): a systematic review and meta-ethnography of qualitative studies. *BMJ Open* 2017;7(1):e012633. doi: 10.1136/bmjopen-2016-012633 [published Online First: 20170113]
25. Brigden A, Shaw A, Anderson E, et al. Chronic fatigue syndrome/myalgic encephalomyelitis in children aged 5 to 11 years: A qualitative study. *Clin Child Psychol Psychiatry* 2021;26(1):18-32. doi: 10.1177/1359104520964528 [published Online First: 20201022]
26. Brigden A, Shaw A, Barnes R, et al. "The child's got a complete circle around him". The care of younger children (5-11 years) with CFS/ME. A qualitative study comparing families', teachers' and clinicians' perspectives'. *Health Soc Care Community* 2020;28(6):2179-89. doi: 10.1111/hsc.13029 [published Online First: 20200609]
27. Clery P, Linney C, Parslow R, et al. The importance of school in the management of Myalgic Encephalomyelitis/Chronic Fatigue Syndrome (ME/CFS): issues identified by adolescents and their families. *Health Soc Care Community* 2022;30(6):e5234-e44. doi: 10.1111/hsc.13942 [published Online First: 20220822]
28. Simila WA, Nost TH, Helland IB, et al. Factors related to educational adaptations and social life at school experienced by young people with CFS/ME: a qualitative study. *BMJ Open* 2021;11(11):e051094. doi: 10.1136/bmjopen-2021-051094 [published Online First: 20211118]
29. Coyne IT. Sampling in qualitative research. Purposeful and theoretical sampling; merging or clear boundaries? *J Adv Nurs* 1997;26(3):623-30. doi: 10.1046/j.1365-2648.1997.t01-25-00999.x
30. Braun V, Clarke V. One size fits all? What counts as quality practice in (reflexive) thematic analysis? *Qualitative Research in Psychology* 2021;18(3):328-52. doi: 10.1080/14780887.2020.1769238
31. Ziebland S, McPherson A. Making sense of qualitative data analysis: an introduction with illustrations from DIPEx (personal experiences of health and illness). *Med Educ* 2006;40(5):405-14. doi: 10.1111/j.1365-2929.2006.02467.x
32. Maclean A, Sweeting H, Hunt K. 'Rules' for boys, 'guidelines' for girls: Gender differences in symptom reporting during childhood and adolescence. *Soc Sci Med* 2010;70(4):597-604. doi: 10.1016/j.socscimed.2009.10.042 [published Online First: 20091122]
33. Harland MR, Parslow RM, Anderson N, et al. Paediatric chronic fatigue syndrome patients' and parents' perceptions of recovery. *BMJ Paediatr Open* 2019;3(1):e000525. doi: 10.1136/bmjpo-2019-000525 [published Online First: 20191202]
34. Parslow R, Patel A, Beasant L, et al. What matters to children with CFS/ME? A conceptual model as the first stage in developing a PROM. *Arch Dis Child* 2015;100(12):1141-7. doi: 10.1136/archdischild-2015-308831 [published Online First: 20151009]
35. Min M, Hancock DG, Aromataris E, et al. Experiences of living with juvenile idiopathic arthritis: a qualitative systematic review. *JBI Evid Synth* 2022;20(1):60-120. doi: 10.11124/JBIES-21-00139
36. Rasalingam A, Fegran L, Brekke I, et al. Young people with long-term health challenges experiences in transition to adulthood: A qualitative metasynthesis. *J Adv Nurs* 2021;77(2):595-607. doi: 10.1111/jan.14641 [published Online First: 20201127]
37. Hopkins L, Green J, Henry J, et al. Staying engaged: the role of teachers and schools in keeping young people with health conditions engaged in education. *Australian Journal of Educational Research* 2014:25-41.

For peer review only

38. Loades ME, Crawley E, Chalder T, et al. Paediatric chronic fatigue syndrome: 25 year perspective. *Clin Child Psychol Psychiatry* 2021;26(1):8-17. doi: 10.1177/1359104520978461 [published Online First: 20201210]

For peer review only

Supplementary file – interview guides

Interview Guide for Experiences of Long Covid in Families study – parents & young people who have had Long Covid themselves (patients)

*NB. Participants may occupy dual roles and be both **carers** and **patients**. Topic guide can be modified accordingly.*

PREAMBLE for young people (who have had Long Covid themselves)
PART ONE: INVITATION TO TELL THEIR OWN STORY OF LONG COVID IN THE FAMILY

For peer review only

In this interview, we would like to hear about your experiences of how Long Covid has affected you and your family. I am interested in whatever you would like to tell me about your experiences (e.g. how this affected where you were living and working/studying and any other health problems you were dealing with) but please do feel free to take a break whenever you need to – we can continue another day if you get tired.

In the first part of the interview I will ask you to tell me about your experiences. After you have finished your story, I will ask you a few extra questions if that's OK.

PART TWO: FOLLOW-UP PROMPTS TO COVER ANY TOPICS NOT COVERED IN INTERVIEWEE'S NARRATIVE - PARENTS AND YOUNG PEOPLE

Initial awareness of Long Covid and ideas about own/ family vulnerability
The purpose of this area of questioning is to develop an understanding of how Long Covid became a concern for the participant and their family and if they made any changes in response (beyond those already in place around Covid)

First awareness of Long Covid
When did you first hear about Long Covid? Can you remember how you heard about it and what you thought it meant? Where did your information come from?

Did you have particular worries or concerns about yourself or members of your family and those close to you?

For example, did you hear anything in the media about some groups of people being more affected by Long Covid than others? How did you feel about that?

Did the awareness of Long Covid lead you to you make any additional changes (i.e. changes beyond those you might have already made in relation to COVID-19) to your daily life or to the people you would see?

Did other people in your family make changes as well? How did that work?

<u>Experiences of becoming ill with Long Covid</u>

Suspecting Long Covid

When did you realise that you might have Long Covid? *(NB use terminology that participant has used to this point)* What was your life like at that time? (school life, work life, social life, family life)

Which month and year was it when you had COVID-19, and then when did you realise it was Long Covid?

If diagnosed: How did you feel about getting diagnosed with Long Covid? Has this changed over time?

Illness experience

(How) has your experience of having Long Covid changed over time? (Prompts: *How) have the symptoms changed over time? Are there any triggers that make your symptoms worse? Is there anything that helps with your symptoms? What have you tried? What happened when you tried this? What has been suggested by others? Are there any symptoms that particularly worry you – either now or thinking about the longer term?*)

(*If relevant – may have been previously fit and well*) Thinking about the health problems or any health conditions you had before you developed Long COVID, have any of these have changed (got worse/better)

Who did you tell when you first had/thought you had Long Covid? (did you tell people you have contact with regularly? What were their responses?)

Actions taken

Have you been able to take care of yourself when you were ill? Did anyone else help care for you?

How did you do to manage in your day-to-day life when you developed Long Covid? Did you make any changes to your usual routines? Did any family members have to make changes to their daily life?

Were there important things that you weren't able to do while you were ill (work, caring responsibilities, religious activities)? How did you manage those things?

Family life

How has long covid affected your day-to-day life? (*prompts: work, relationships, ability to do things you previously enjoyed, sleep etc*)

Can you tell me about how Long Covid has affected your family life?

For peer review only

Have there been any disruptions to key tasks in your family life e.g. to childcare, household routines, work, shopping, family-owned or run businesses etc.

If split or multiple households: can you tell me about how you've managed across households? How about with extended family?

Have there been any changes in your family roles? (*prompts: who works, childcare, household tasks)*

What was your day like yesterday? OR Talk me through what a typical day looks like for your family. Have there been times when it was more difficult? What has made it easier?

Have you had to deal with anything similar to this before as a family? *If yes:* can you tell me about your earlier experiences?

If relevant: Do you have any concerns about regaining your independence / regaining normality?

Is there anything you continue to struggle with as a family? How does this make you feel about the future?

Are there any other ways in which having Long Covid is still affecting your day-to-day family life or your plans for the future? (use prompts as necessary)

- Impact on work school and other daily activities
- Impact on family and social life
- Returning to work/getting a new job (support from employer/colleagues, Occupational Health, adjustments, changed hours and positive and negative experiences)
- Financial issues (were you working, furloughed, did you get sick leave, did you get sick pay, state benefits, travel costs, insurances, change in outgoings, for younger people: statement of special educational needs, disabled students allowance)
- Things you've had to cancel, abandon or postpone

Was there anything you felt was particularly unfair about your family's experience of Long Covid?

Information and care-seeking

How was the communication with your GP? What went well? What didn't go well? Talk me through what the conversation was like.

Can we talk a bit more about info about LC?

What are the main sources of information that you have accessed or made use of in relation to Long Covid? What if anything has been helpful/unhelpful about this information?

For peer review only

To what extent do you think Long Covid is well understood? (by those around you, healthcare staff, employers)

Accessing healthcare for LC

How did you come to understand that you have LC?
Have you seen your GP about your LC?

Can you talk me through how you've found accessing healthcare for LC?

What happened? (probe for both helpful and unhelpful responses)?

- And talking to the GP receptionist when you booked, how was that?

(if there was a Long Covid diagnosis) How was your diagnosis of Long Covid communicated to you?

- Talk us through what the conversation was like?
- How did you feel about that?
- What can you remember about the types of things your GP said?

How often do you talk to your GP since your diagnosis?

- Do you speak to the same GP?
- If it varies: how come?

Has there been any changes to the discussion of long Covid with your GP since diagnosis?

- Talk us through the changes?

What if anything do you talk to your GP about in terms of your family situation and the impact of long Covid?

Have you had any referral or any treatment/advice offered for long Covid?

- How were they offered?

  If dietary supplements, rest or graded exercise or PACE (progressive accelerating cardiopulmonary exertion) are mentioned; please ask how these were explained and how the conversation went.

What, if any, concerns about Long Covid did you talk to your GP about?

- How were these concerns responded to?

Is there anything your GP said that was helpful/unhelpful?

We will be using these interviews to develop training for GPs about how to talk to patients with LC? DO you have any recommendations for this training, based on what has happened to you?

Have you been referred to or referred yourself to any services specially designed for people with Long Covid? *Tell us all about it! When, how often, what happened, any surprises, when you are going back etc etc*

For peer review only

Self-management for LC

Have you taken any medicine, over the counter remedies or anything else to manage your LC symptoms, either ones prescribed for you or ones you have bought/used yourself (*might include pain killers, other over-the-counter medications for fever/pain control)?*

Have you taken any nutritional supplements, vitamins, particular foods or changed your diet at all in relation to Long Covid? What (why, who) prompted you to make this change? Has it helped?

Have you used any complementary / alternative approaches (what, why, attitude to)?

Recovery

Would you say that you have 'recovered' from having Long Covid or that you feel like you're in the process of recovering? If 'recovered'/recovering

- When (how long after first symptoms) did you recognise or feel confident that you were getting better? Did some aspects of your health improve quicker? Did some improve more slowly?
- Were any aspects of your process of recovering from Long Covid particularly encouraging or frustrating?
- What do you think has helped you to recover?
- Were there any things that you think slowed your recovery?
- Is there any advice that you think could be helpful in speeding or supporting the recovery of others with Long Covid?

Do you feel protected from catching COVID-19 again?

Have you had a COVID-19 vaccination?
(*If had vaccination)* How was your experience of vaccination (which vaccine did you have? When? Did you experience side effects? Did you notice any change in your LC symptoms after vaccination?)

Closing questions/reflections

Service improvement

There is still a lot that is unknown about LC – what are the questions that you would like to see answered in the future?

Do you have any messages for others affected by the condition (including advice on what they might talk to their doctors about)

(To ask those who had some interaction with some part of the health service):
People who have been through treatment in the NHS often notice things that don't seem to make sense, or seem inefficient or confusing. Sometimes they have really good ideas about

For peer review only

what might improve things. Is there anything you noticed that you think could/ should be changed to improve how people with Long Covid are treated and supported to recover?

What services aren't provided that you'd like to see?

Could services be better organised?

Do you think there are some services you don't need or services which could be replaced with something else?

What's the one change that you would most like to see? If you were asked to say what good care for someone with your condition looks like, what would you say?

Or what are the features of really good care for someone with Long Covid?

Is there anything else you would like to talk about?

Interview Guide for Experiences of Long Covid in Families study – parents or young people (carers of someone with Long Covid)

NB. Participants may occupy dual roles and be both carers and patients. Topic guide can be modified accordingly.

PREAMBLE for parents (who are caring for a child with Long Covid)

PART ONE: INVITATION TO TELL THEIR OWN STORY OF LONG COVID IN THE FAMILY

In this interview, we would like to hear about your experiences of how Long Covid has affected you and your family. I am interested in whatever you would like to tell me about your experiences but please do feel free to take a break whenever you need to – we can continue another day if you get tired.

In the first part of the interview I will ask you to tell me about your family's and your child's experiences.

After you have finished your story, I will ask you a few extra questions if that's OK.

PART TWO: FOLLOW-UP PROMPTS TO COVER ANY TOPICS NOT COVERED IN INTERVIEWEE'S NARRATIVE - PARENTS AND YOUNG PEOPLE CARING FOR A FAMILY MEMBER

Initial awareness of Long Covid and ideas about own/ family vulnerability

The purpose of this area of questioning is to develop an understanding of how Long Covid became a concern for the participant and their family and if they made any changes in response (beyond those already in place around Covid)

First awareness of Long Covid

For peer review only

When did you first hear about Long Covid? Can you remember how you heard about it and what you thought it meant? Where did your information come from?

Did you have particular worries or concerns about yourself or members of your family and those close to you?

For example, did you hear anything in the media about some groups of people being more affected by Long Covid than others? How did you feel about that?

Did the awareness of Long Covid lead you to you make any additional changes (i.e. changes beyond those you might have already made in relation to COVID-19) to your daily life or to the people you would see?

Did other people in your family make changes as well? How did that work?

<u>Experiences of family member becoming ill with Long Covid</u>

Suspecting Long Covid

When did [family member] realise that they might have Long Covid? *(NB use terminology that participant has used to this point) OR*
When did you realise that [family member] might have Long Covid?
What was your/their life like at that time? (school life, work life, social life, family life)

Which month and year was it when they had COVID-19, and then when did you/they realise it was Long Covid?

If diagnosed: How did you feel about [family member] getting diagnosed with Long Covid? How did they feel about it? Has this changed over time?

Illness experience

(How) has [family member's] experience of having Long Covid changed over time? (Prompts: *How) have the symptoms changed over time? Are there any triggers that make their symptoms worse? Is there anything that helps with their symptoms? What have you/they tried? What happened when you/they tried this? What has been suggested by others? Are there any symptoms that particularly worry you or them – either now or thinking about the longer term?*)

(*If relevant – may have been previously fit and well*) Thinking about the health problems or any health conditions [family member] had before they developed Long COVID, have any of these have changed (got worse/better)

Who did you tell when you first had/thought [family member] had Long Covid? (did you tell people you have contact with regularly? What were their responses?)

Actions taken

Has anyone else helped care for/look after [family member] with you?

How did you do to manage in your day-to-day life when [family member] developed Long Covid? Did you make any changes to your usual routines? Did any other family members have to make changes to their daily life?

Were there important things that you weren't able to do while you were looking after [family member] (work, other caring responsibilities, religious activities)? How did you manage those things?

Family life

How has [family member's] long covid affected your day-to-day life? (*prompts: work, relationships, ability to do things you previously enjoyed, sleep etc*)

Can you tell me about how Long Covid has affected your family life?

Have there been any disruptions to key tasks in your family life e.g. to childcare, household routines, work, shopping, family-owned or run businesses etc.

If split or multiple households: can you tell me about how you've managed across households? How about with extended family?

Have there been any changes in your family roles? (*prompts: who works, childcare, household tasks)*

What was your day like yesterday? OR Talk me through what a typical day looks like for your family. Have there been times when it was more difficult? What has made it easier? Have you had to deal with anything similar to this before as a family? *If yes:* can you tell me about your earlier experiences?

If relevant: Do you have any concerns about regaining your independence / regaining normality?

Is there anything you continue to struggle with as a family? How does this make you feel about the future?

Are there any other ways in which having Long Covid is still affecting your day-to-day family life or your plans for the future? (use prompts as necessary)

- Impact on work school and other daily activities
- Impact on family and social life
- Returning to work/getting a new job (support from employer/colleagues, Occupational Health, adjustments, changed hours and positive and negative experiences)
- Financial issues (were you working, furloughed, did you get sick leave, did you get sick pay, state benefits, travel costs, insurances, change in outgoings,

For peer review only

for younger people: statement of special educational needs, disabled students allowance)
- Things you've had to cancel, abandon or postpone

Was there anything you felt was particularly unfair about your family's experience of Long Covid?

<u>Information and care-seeking</u>

How was the communication with your GP? What went well? What didn't go well? Talk me through what the conversation was like.

Can we talk a bit more about info about LC?
What are the main sources of information that you have accessed or made use of in relation to Long Covid? What if anything has been helpful/unhelpful about this information?

To what extent do you think Long Covid is well understood? (by those around you, healthcare staff, employers)

Accessing healthcare for LC

How did you/[family member] come to understand that they have LC?
Has [family member] seen their GP about their LC? Did you go with them?

Can you talk me through how [family member] has found accessing healthcare for LC? *OR*

Can you talk me through how you've found accessing care for [family member's] LC?

What happened? (probe for both helpful and unhelpful responses)?

- And talking to the GP receptionist when you/they booked, how was that?

(if there was a Long Covid diagnosis) How was the diagnosis of Long Covid communicated to you?

- Talk us through what the conversation was like?
- How did you feel about that?
- What can you remember about the types of things the GP said?

How often do you talk to the GP since the diagnosis?

- Do you speak to the same GP?
- If it varies: how come?

Has there been any changes to the discussion of long Covid with the GP since diagnosis?

- Talk us through the changes?

What if anything do you talk to the GP about in terms of your family situation and the impact of long Covid?

Has [family member] had any referral or any treatment/advice offered for long Covid?

For peer review only

- How were they offered?
    If dietary supplements, rest or graded exercise or PACE (progressive accelerating cardiopulmonary exertion) are mentioned; please ask how these were explained and how the conversation went.

What, if any, concerns about Long Covid did you talk to the GP about?

- How were these concerns responded to?

Is there anything the GP said that was helpful/unhelpful?

We will be using these interviews to develop training for GPs about how to talk to patients with LC? DO you have any recommendations for this training, based on what has happened to your family?

Has [family member] been referred to or referred themselves to any services specially designed for people with Long Covid? *Tell us all about it! When, how often, what happened, any surprises, when you are going back etc etc*

Self-management for LC

Has [family member] taken any medicine, over the counter remedies or anything else to manage their LC symptoms, either ones prescribed for them or ones you/they have bought/used yourself (*might include pain killers, other over-the-counter medications for fever/pain control)?*

Have they taken any nutritional supplements, vitamins, particular foods or changed their diet at all in relation to Long Covid? What (why, who) prompted you/them to make this change? Has it helped?

Have you/they used any complementary / alternative approaches (what, why, attitude to)?

Recovery

Would you say that [family member] has ‘recovered’ from having Long Covid or that you feel like they’re in the process of recovering? If ‘recovered’/recovering

- When (how long after first symptoms) did you recognise or feel confident that they were getting better? Did some aspects of their health improve quicker? Did some improve more slowly?
- Were any aspects of the process of recovering from Long Covid particularly encouraging or frustrating?
- What do you think has helped them to recover?
- Were there any things that you think slowed the recovery?
- Is there any advice that you think could be helpful in speeding or supporting the recovery of others with Long Covid?

Do you feel that [family member] is protected from catching COVID-19 again?

If relevant: Has [family member] had a COVID-19 vaccination?

For peer review only

(*If had vaccination)* How was you're the experience of vaccination (which vaccine did they have? When? Did they experience side effects? Did you notice any change in you're their LC symptoms after vaccination?

Closing questions/reflections

Service improvement

There is still a lot that is unknown about LC – what are the questions that you would like to see answered in the future?

Do you have any messages for others affected by the condition (including advice on what they might talk to their doctors about)

(To ask those who had some interaction with some part of the health service):

People who have been through treatment in the NHS often notice things that don't seem to make sense, or seem inefficient or confusing. Sometimes they have really good ideas about what might improve things. Is there anything you noticed that you think could/ should be changed to improve how people with Long Covid are treated and supported to recover?

What services aren't provided that you'd like to see?

Could services be better organised?

Do you think there are some services you don't need or services which could be replaced with something else?

What's the one change that you would most like to see? If you were asked to say what good care for someone with your condition looks like, what would you say?

Or what are the features of really good care for someone with Long Covid?

Is there anything else you would like to talk about?

For peer review only

Standards for Reporting Qualitative Research (SRQR)*

http://www.equator-network.org/reporting-guidelines/srqr/

Title and abstract

	Page/line no(s).
**Title** - Concise description of the nature and topic of the study Identifying the study as qualitative or indicating the approach (e.g., ethnography, grounded theory) or data collection methods (e.g., interview, focus group) is recommended	1
**Abstract** - Summary of key elements of the study using the abstract format of the intended publication; typically includes background, purpose, methods, results, and conclusions	2

Introduction

**Problem formulation** - Description and significance of the problem/phenomenon studied; review of relevant theory and empirical work; problem statement	3
**Purpose or research question** - Purpose of the study and specific objectives or questions	3

Methods

**Qualitative approach and research paradigm** - Qualitative approach (e.g., ethnography, grounded theory, case study, phenomenology, narrative research) and guiding theory if appropriate; identifying the research paradigm (e.g., postpositivist, constructivist/ interpretivist) is also recommended; rationale**	3-4
**Researcher characteristics and reflexivity** - Researchers' characteristics that may influence the research, including personal attributes, qualifications/experience, relationship with participants, assumptions, and/or presuppositions; potential or actual interaction between researchers' characteristics and the research questions, approach, methods, results, and/or transferability	4
**Context** - Setting/site and salient contextual factors; rationale**	4
**Sampling strategy** - How and why research participants, documents, or events were selected; criteria for deciding when no further sampling was necessary (e.g., sampling saturation); rationale**	4
**Ethical issues pertaining to human subjects** - Documentation of approval by an appropriate ethics review board and participant consent, or explanation for lack thereof; other confidentiality and data security issues	4
**Data collection methods** - Types of data collected; details of data collection procedures including (as appropriate) start and stop dates of data collection and analysis, iterative process, triangulation of sources/methods, and modification of procedures in response to evolving study findings; rationale**	4

For peer review only

**Data collection instruments and technologies** - Description of instruments (e.g., interview guides, questionnaires) and devices (e.g., audio recorders) used for data collection; if/how the instrument(s) changed over the course of the study	4
**Units of study** - Number and relevant characteristics of participants, documents, or events included in the study; level of participation (could be reported in results)	4-6
**Data processing** - Methods for processing data prior to and during analysis, including transcription, data entry, data management and security, verification of data integrity, data coding, and anonymization/de-identification of excerpts	4
**Data analysis** - Process by which inferences, themes, etc., were identified and developed, including the researchers involved in data analysis; usually references a specific paradigm or approach; rationale**	4
**Techniques to enhance trustworthiness** - Techniques to enhance trustworthiness and credibility of data analysis (e.g., member checking, audit trail, triangulation); rationale**	4

Results/findings

**Synthesis and interpretation** - Main findings (e.g., interpretations, inferences, and themes); might include development of a theory or model, or integration with prior research or theory	6-8
**Links to empirical data** - Evidence (e.g., quotes, field notes, text excerpts, photographs) to substantiate analytic findings	6-8

Discussion

**Integration with prior work, implications, transferability, and contribution(s) to the field** - Short summary of main findings; explanation of how findings and conclusions connect to, support, elaborate on, or challenge conclusions of earlier scholarship; discussion of scope of application/generalizability; identification of unique contribution(s) to scholarship in a discipline or field	8-10
**Limitations** - Trustworthiness and limitations of findings	

Other

**Conflicts of interest** - Potential sources of influence or perceived influence on study conduct and conclusions; how these were managed	10
**Funding** - Sources of funding and other support; role of funders in data collection, interpretation, and reporting	10

*The authors created the SRQR by searching the literature to identify guidelines, reporting standards, and critical appraisal criteria for qualitative research; reviewing the reference lists of retrieved sources; and contacting experts to gain feedback. The SRQR aims to improve the transparency of all aspects of qualitative research by providing clear standards for reporting qualitative research.

For peer review only

**The rationale should briefly discuss the justification for choosing that theory, approach, method, or technique rather than other options available, the assumptions and limitations implicit in those choices, and how those choices influence study conclusions and transferability. As appropriate, the rationale for several items might be discussed together.

Reference:

O'Brien BC, Harris IB, Beckman TJ, Reed DA, Cook DA. **Standards for reporting qualitative research: a synthesis of recommendations.** *Academic Medicine*, Vol. 89, No. 9 / Sept 2014 DOI: 10.1097/ACM.0000000000000388

For peer review only
